# Standalone Integrated Magnonic Devices

**DOI:** 10.1002/adma.202503493

**Published:** 2025-07-23

**Authors:** M. Cocconcelli, F. Maspero, A. Micelli, A. Toniato, A. Del Giacco, N. Pellizzi, A. E. Plaza, A. Cattoni, M. Madami, R. Silvani, C. Adelmann, A. A. Hamadeh, P. Pirro, S. Tacchi, F. Ciubotaru, R. Bertacco

**Affiliations:** ^1^ Dipartimento di Fisica Politecnico di Milano Via G. Colombo 81 Milano 20133 Italy; ^2^ Dipartimento di Fisica e Geologia Università di Perugia Via A. Pascoli Perugia 06123 Italy; ^3^ Fachbereich Physik and Landesforschungszentrum OPTIMAS Rheinland‐Pfälzische Technische Universität Kaiserslautern‐Landau 67663 Kaiserslautern Germany; ^4^ Istituto Officina, dei Materiali del CNR (CNR‐IOM) Unità di Perugia c/o Dipartimento di Fisica e Geologia Università di Perugia Via A. Pascoli Perugia 06123 Italy; ^5^ imec Kapeldreef 75, Heverlee Leuven 3001 Belgium

**Keywords:** magnonic devices, permanent micromagnets, phase shifter

## Abstract

In the race toward “beyond 6G” telecommunication platforms, magnonics emerges as a promising solution. To date, however, the requirement for bulky external sources of the magnetic bias field necessary for spin wave propagation has constituted a significant bottleneck, impeding the integration of magnonic devices into RF systems. Here, the first demonstration is presented of a standalone and tunable magnonic device featuring all‐electric input and output, fully integrated on a silicon substrate, with a compact footprint of 100 × 150 µm^2^. The device consists of a CoFeB waveguide equipped with two radio frequency antennas, flanked by a symmetric configuration of magnetic flux concentrators and SmCo permanent micromagnets. By varying the distance *D* between the flux concentrators and the permanent magnets from 0 to 12 µm, the transverse bias field can be tuned from 20.5 to 11 mT, respectively. This variation directly modulates the dispersion relation of Damon‐Eshbach spin wave modes in the CoFeB waveguide. In the proof‐of‐concept devices, the spin wave frequency band ranges from 3 to 8 GHz, with precise tuning of the phase shift up to 120 deg at 6 GHz. The operational frequency band can be easily pushed to higher frequencies through micromagnet engineering.

## Graphical abstract

A standalone (not requiring external bias magnetic field) and tunable magnonic device is reported with all‐electric input and output, fully integrated on silicon (total footprint of 100 × 150 µm) and 3–8 GHz frequency band. This is achieved by monolithic integration of a CoFeB conduit with RF antennas and an assembly of NiFe and SmCo micromagnets providing a tunable bias field.

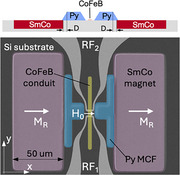

## 1 Introduction

To date magnonics, the study of spin waves (SWs) in magnetic media, has remained primarily a topic of academic research, focused on the unique properties of these excitations in ferro‐, ferri‐, and antiferromagnetic materials.^[^ ^1^, ^2^, ^3^, ^4^ ^]^ Unlike other types of waves, spin waves band‐dispersion can be easily tuned by setting the relative orientation between the magnetic bias field (**H_0_**) and the wave vector (**k**) which defines the different configurations: “Forward Volume – FW” (**H_0_** out‐of‐plane, **k** in‐plane), “Backward volume – BV” (**H_0_** // **k**, in‐plane), “Damon Eshbach – DE” (**H_0_** ⊥ **k**, in‐plane).^[^ ^5^ ^]^ Within each configuration, additional tunability is achieved by varying the magnitude of **H₀**, making spin waves a natural platform for reconfigurable devices. Beyond the linear regime, SW display intriguing non‐linear processes connected to multi‐magnon scattering.^[^ ^6^, ^7^, ^8^, ^9^, ^10^, ^11^ ^]^ Bose condensation (BC) in BV configurations has been reported and used to investigate intriguing properties of BC states as well implement the classical analog of qubit logic.^[^ ^12^, ^13^ ^]^ Furthermore, the interaction between SW and magnetic media can be exploited to move domain walls and reconfigure memory/logic elements.^[^ ^14^ ^]^ Finally, SW “naturally” provides a viable alternative to acoustic waves for “Beyond 6G” applications. Surface and bulk acoustic wave technology is facing challenges in covering the demands of a reliable operation over the whole frequency range involved while maintaining reasonable insertion losses, reduced complexity, and low power dissipation. Spin waves, instead, can cover a broad spectrum, ideally from a few MHz up to 1 THz. This makes them a promising candidate for the implementation of high‐frequency communication technology.^[^ ^15^, ^16^, ^17^, ^18^ ^]^

Despite these advantages, just a few examples of magnonic applications are currently available in the market. Apart from Yttrium‐Iron‐Garnet (YIG) tuned RF oscillators and Auto‐tune filters used to mitigate electro‐magnetic interference (e.g., by Metamagnetics Inc.) there are no real implementations of exciting devices reported in literature such as RF filters, phase‐shifters, interferometers, spectrum analyzers, etc. The main reason for this is twofold: i) many proposed devices are based on epitaxial YIG, providing the longest propagation length but not suitable for integration with Silicon electronics,^[^ ^19^ ^]^ ii) to operate within the frequency bands required for typical applications (above 4–5 GHz), magnonic devices necessitate an external magnetic bias field usually produced by bulky electromagnets which are unsuitable for integration into consumer electronic devices.

On the other hand, it has been shown that electric fields can be used to modify the internal magnetic field either via the magnetoelectric effect in multiferroic systems or through interfacial voltage‐controlled magnetic anisotropy (VCMA). Although the magnetoelectric effect offers a promising route for tuning the dispersion relation of spin waves, direct modulation of spin wave phase via this mechanism is still in the early stages of development.^[^ ^20^, ^21^ ^]^ In contrast, VCMA has gained significant traction, particularly in ultrathin ferromagnetic films where it enables low‐power manipulation of magnetic anisotropy. However, this interfacial effect is typically confined to nanometer‐thick layers, where spin wave propagation is strongly attenuated due to reduced group velocity, whereas the transmitted energy is limited by the reduced magnetic volume.^[^ ^22^, ^23^ ^]^ Another interesting route for tuning SW propagation is that based on the application of strain to the magnetic medium, thus generating a local effective magnetic field via magnetostriction.^[^ ^24^ ^]^ However, this approach does not fix the problem of the generation of the external bias field necessary to stabilize the targeted configuration for SW (DE, FW, BV) that can then be finely tuned via magnetostriction.

In this work, we address some relevant challenges of magnonics by demonstrating the first standalone, monolithically integrated magnonic device on silicon that operates without an external magnetic field and is suitable for integration into portable electronic platforms such as mobile phones. While prior studies have reported zero‐field SW propagation in nanostructures relying on shape anisotropy, and other works have demonstrated RF devices integrated with external magnets, to the best of our knowledge, this is the first demonstration of a silicon‐integrated magnonic device operating at frequencies up to 8 GHz.^[^ ^25^, ^26^ ^]^

Our concept is based on the exploitation of a suitable combination of permanent micro‐magnets and magnetic flux concentrators (MFCs), fabricated on the same silicon chip hosting the magnonic waveguide, to produce a bias field up to 20.5 mT which is strong enough to stabilize a DE configuration. Varying the distance (D) between the permanent micromagnets and the MFC we can tune the bias field in the 11–20.5 mT range so that our first proof‐of‐concept device behaves like a tunable phase‐shifter (or time delay unit) in the 3–8 GHz range, even though the frequency band can be easily shifted upward by proper engineering of the permanent magnets and MFCs. The device is equipped with two (input – output) RF antennas for a full electric operation of the device whose total footprint (100 × 150 µm) makes it compatible with a system‐on‐chip or monolithic integration with CMOS electronics.

This advancement is made possible by a custom planar process that enables monolithic integration of unconventional functional materials on silicon, despite their differing fabrication requirements. These include hard magnets like SmCo, providing a stable bias magnetic field due to its high coercivity exceeding 2 T; soft magnetic multilayers such as NiFe/Cr, which facilitate efficient magnetic flux guidance owing to their high permeability (>1000); and low‐damping ferromagnets like CoFeB, used for effective spin wave propagation. Note that, while rare‐earth magnets like SmCo and NdFeB are commonly employed in bulk applications such as motors and actuators, this work marks the first integration of such materials into an RF device on silicon.

## 2 Results

### 2.1 Chip Layout

**Figure 1:**
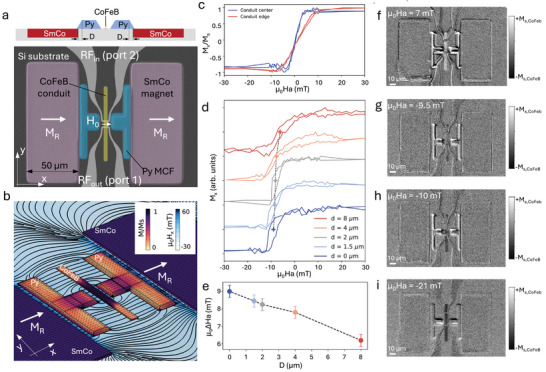
a). False‐color SEM image and schematic cross section of a device. b) COMSOL Multiphysics simulation of the stray field H produced by the assembly of SmCo permanent magnets and MFC made of Permalloy (field lines and blue color scale for the H_x_ component), combined with the simulation of the micromagnetic configuration of the CoFeB conduit (arrows and brown color scale for the M_x_ component). c) Mx versus Hx loops measured at the center (within the poles of the MFC) and edge of the CoFeB conduit, prior to the initial magnetization at 2T of the SmCo magnets. d) Loops measured at the center of the conduit after magnetization of the SmCo micromagnets at 2T, in devices with different distances *D* between the MFC and the SmCo magnets. e) Shift ΔHa of the loops reported in panel d for various values of D. f–i) Snapshots from a video (available as Supplementary Material) taken with a microMOKE apparatus during a whole loop, at characteristic applied fields µ_0_Ha = +7, −9.5, −10, −21 mT, respectively.

**Figure** **1**a shows a false‐color SEM image of our device. The green vertical rectangle represents the CoFeB waveguide (25 nm thick, 3.6 µm wide) for SW propagation, with two inductive antennas (grey) made of gold (125 nm thick, 1 µm wide) above the CoFeB for the input and output RF signals. Between the antennas and the CoFeB conduit, there is a silica spacer with a thickness of ≈70 nm. The central region of the conduit, with the two antennas at 5 µm distance (center to center), is flanked by two T‐shaped MFC (blue), made of a Py/Cr multilayer with tapered edges, having 1 µm thickness and 10 µm width (see methods for details). Two SmCo permanent micromagnets (pink rectangles in Figure 1 with 1 µm thickness and 50 × 100 µm footprint) with remanent magnetization µ_0_M_R_ = 0.6 T upon magnetization at 2 T, are placed at a variable distance *D* (0, 1.5, 4, 8, 12 µm) from the MFC in order to tune the coupling between them and, consequently, the field H_0_ produced in the CoFeB region between the antennas.^[^ ^27^, ^28^, ^29^, ^30^ ^]^ SmCo was chosen due to its very high coercivity (>2 T), ensuring that the magnetic field produced in remanence is stable against external perturbations, combined with good remanence and high potential for monolithic integration on CMOS. Even though in this paper, due to some technical limitations of the available sputtering machine, we use SmCo layers grown without substrate heating and then requiring a post‐annealing at 650 °C to develop the hard‐phase, there is evidence in literature for processes directly achieving the hard‐phase during growth at 350 °C,^[^ ^31^ ^]^ i.e., with a thermal budget compatible with CMOS technology. This is not the case of the other widely used rare‐earth magnet (NdFeB), which requires a much higher thermal budget and then seems less suitable for integration. The micromagnets serve as the on‐chip source of the magnetic field, while the MFCs concentrate the generated field between their poles, enhancing both its intensity and uniformity in that region. The MFC action is visible in Figure 1b, showing the results of a COMSOL simulation of the static magnetic field for a distance *D* = 4 µm. More details on the field profiles can be found in Figure S1 (Supporting Information), from which it also emerges that the stray field produced by SmCo rapidly decreases in the outer region (reaching a vale below 1 mT at 50 µm from the edges of the micromagnets), so that the cross‐talk between adjacent devices like that considered in this paper does not constitute a major issue. In future device layouts, soft magnetic shielding layers could be incorporated using the same fabrication process to further suppress unwanted crosstalk between adjacent devices, should this become necessary. Numerical simulations indicate that the MFC enables field concentration, achieving a gain factor G >2 for an external uniform bias field within the MFC's linear region (≈±5 mT). However, for larger applied fields, G can drop below unity due to partial saturation of the MFC. Notably, the T‐shaped MFCs configuration was selected to maximize the bias magnetic field generated between their poles under the influence of the stray field from the SmCo permanent magnets, which is highly non‐uniform. This choice was made over more conventional trapezoidal designs, which, while offering higher gain for uniform fields, are less effective when coupled with rectangular SmCo magnets (see Section S1, Supporting Information).

We conducted microMOKE experiments to characterize the MFC behavior and estimate the bias field H_0_ produced at the center of the CoFeB waveguide for different distances D. Figure 1c shows the loops measured for *D* = 0 in the pristine state of the SmCo permanent magnet, i.e., prior to their magnetization with a field of 2 T, with an applied transverse field **Ha** along the x direction. Blue and red curves represent, respectively, the loops taken at the center of the CoFeB conduit, in the region flanked by the MFC, and at the top edge, far away from the horizontal arms of the MFC. We observe the typical “loop without hysteresis” expected for a transverse applied field with respect to the easy *y*‐axis determined by shape anisotropy. The curves are quite symmetric with respect to Ha = 0, thus indicating that SmCo magnets in the pristine state do not provide a sizable bias. On the other hand, when moving from the edge to the center, the slope of the linear region increases by a factor of 2.4 ± 0.3 which represents a good estimate of the MFC gain if we assume that the action of the MFC at the conduit edge is almost negligible, as seen in simulations. The situation completely changes upon magnetization of the SmCo magnet along the *x*‐axis with µ_0_Ha = 2 T, the maximum field available in our laboratory, as reported in panel 1d showing loops taken at the conduit center (loops taken at the edge are shown in Section S2, Supporting Information). A true hysteresis loop appears (blue line, corresponding to *D* = 0 µm), shifted by 9 mT toward negative fields because of the bias field from SmCo magnets. The reason for the appearance of hysteresis is connected to domain wall pinning by the non‐uniform bias field in the central region of the conduit, as visible in the sequence of images form a video (Video S1, Supporting Information, available online as supporting material) reported in panels f,g,h,i of Figure 1, corresponding to snapshots taken at +7, −9.5, −10, −21 mT, respectively. We observe that magnetization reversal takes place via nucleation and propagation of domain walls which are pinned by the high field region between the horizontal arms of the MFC (see panel 1 h) thus leading to the characteristic Barkhausen jumps seen in the loops and to hysteresis. Noteworthy, the shift of the loop µ_0_∆Ha decreases from 9 ± 0.2 to 6.2 ± 0.2 mT when increasing *D* due to the less efficient coupling between the stray field from SmCo magnets and MFCs, as summarized in panel 1e, and this provides the way to tune the bias field H_0_. H_0_ is related to, but does not directly correspond to, the loop shift ΔHa due to the gain factor G introduced by the MFCs on the external field. This relationship is given by H_0_ = G⋅∆Ha. The microMOKE measurements thus provide an initial estimate of the effective bias field achievable in our device, ranging from 21.6 ± 3.4 to 15 ± 2.4 mT as *D* increases from 0 to 8 µm.

### 2.2 Broadband Spectroscopy

**Figure 2:**
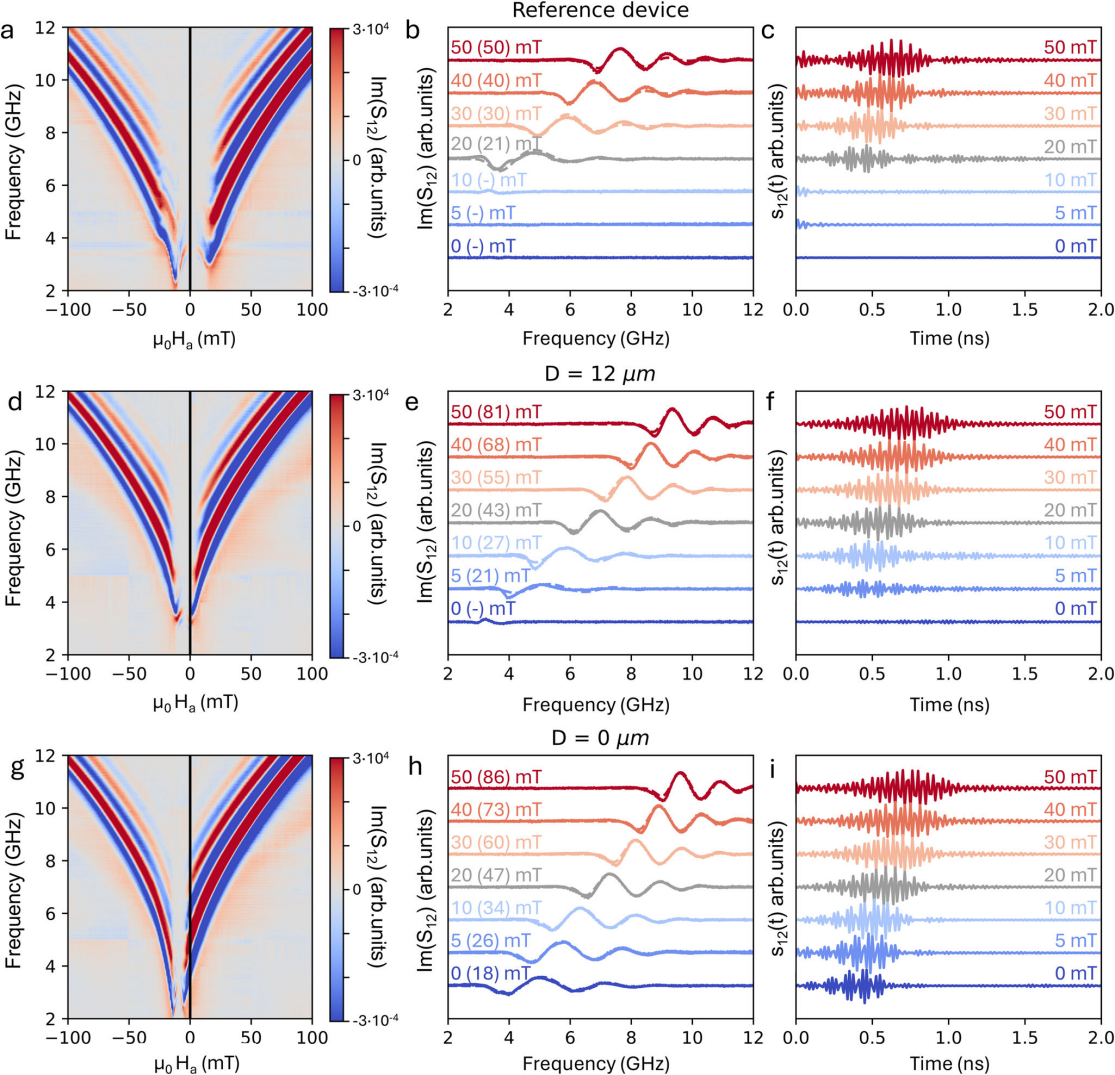
Panels a,b,c report VNA measurements on reference CoFeB conduits without MFC and permanent magnets. a) 2D color map of the oscillations related to the imaginary part of S_12_versus frequency for various applied fields Ha. b) Experimental S_12_(f) curves (continuous lines) for selected values of Ha (numbers in mT outside the brackets close to each curve), together with the result of the fit using Equation (1) (dashed lines) from which the actual values of the effective applied bias field (numbers in brackets) are retrieved. c) Impulse response s_12_(t) obtained from the time‐domain analysis of the S_12_(f) scattering parameters corresponding to the curves in Figure 2b. Panels d–f report the same analysis as in a–c for devices with a distance between MFC and permanent magnets *D* = 12 µm. Panels g,h,i refer to a device with *D* = 0 µm.

**Figure 3:**
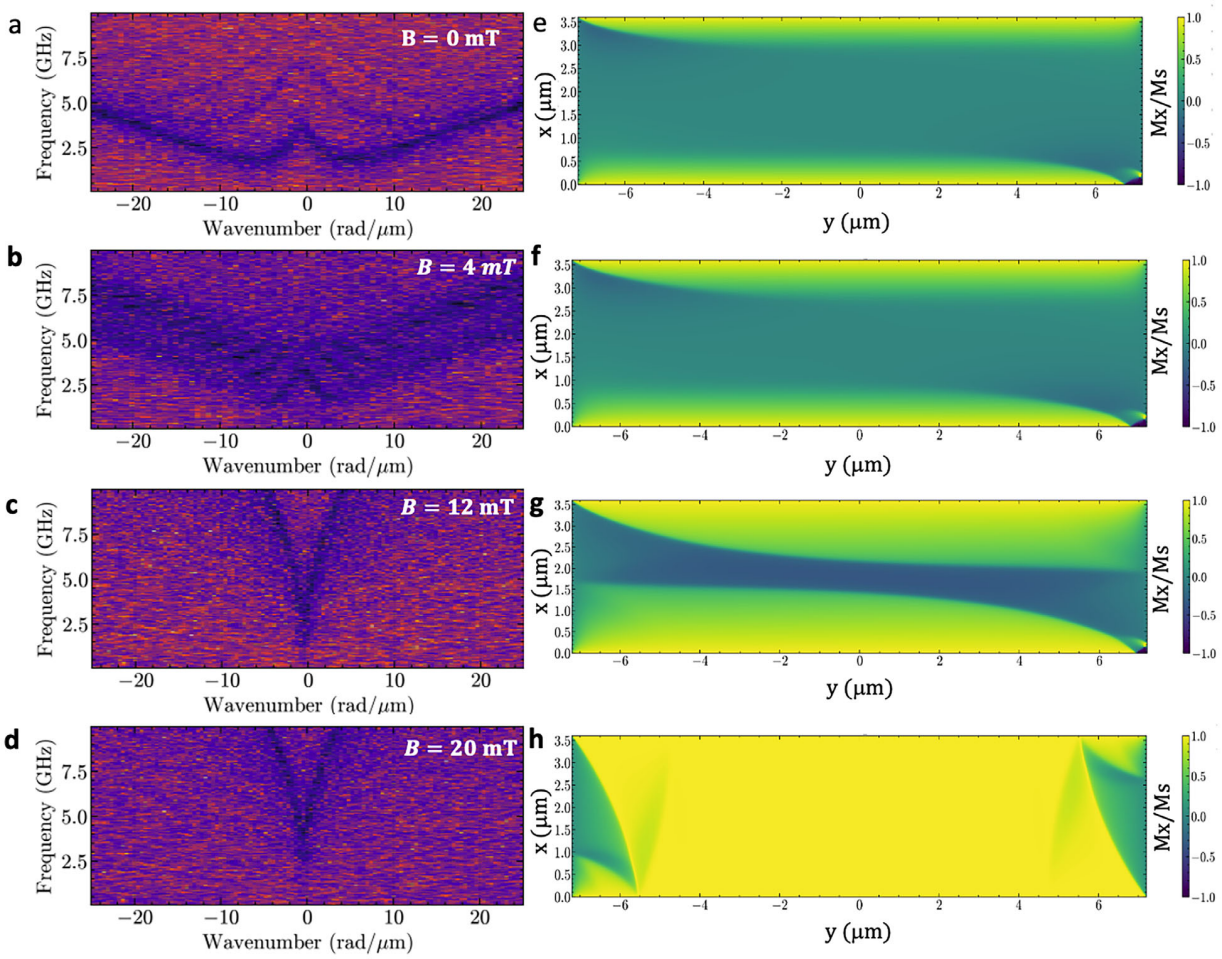
Panels a–d) band dispersion simulated with mumax3 for reference CoFeB conduits at different applied field B = µ_0_Ha. Panels e–h) show the corresponding static micromagnetic configurations. One clearly recognizes the transmission from a multiplicity of BV width modes at low field (a,b) to a single DE mode at higher field (c,d).

We then carried out the investigation of SW propagation in the DE configuration using VNA spectroscopy.^[^ ^32^, ^33^ ^]^ We stress here that, even though the main achievement of this paper is the demonstration of RF magnonic devices working without external applied bias field, we first carried out a full characterization of SW propagation in our devices at variable applied external field. First, we analysed SW transmission in reference devices made of just the CoFeB waveguide and inductive antennas, by measuring the scattering matrix S_ij_ as a function of frequency for different applied transverse fields µ_0_Ha_x_, from −100 to +100 mT in steps of 1 mT. The color plot of the imaginary part of S_12_ versus frequency and field is reported in **Figure** **2**a, after subtraction of a reference signal taken at zero applied field Ha, when no coherent propagation of SW is expected, and noise removal via time gating (details on the signal processing are reported in the methods and in Section S3, Supporting information). At low field the DE configuration is not well defined (see the static micromagnetic configuration of **Figure** **3**) and we do not observe the oscillations in the Im(S_12_(f)) which are the fingerprint of SW propagation. For|µ_0_Ha| >10 mT we start observing some oscillations, but a clear signature of DE modes appears only for |µ_0_Ha| >20 mT, where we see that the lower edge of oscillations (close to the ferromagnetic resonance (FMR) frequency for the conduit at that peculiar field) follows a Kittel‐like trend for a transversally applied external field. This is in nice agreement with the micromagnetic simulations of the conduit without surrounding magnets (see Figure 3), showing that a single domain configuration with transverse magnetization is retrieved only above 20 mT of applied field. The micromagnetic simulations show that at lower fields, the stripe supports many BV width modes (see Figure 3a), which are, however, not efficiently excited since the main dynamic field components of the antenna (pointing along the in‐plane x direction) is parallel to the static magnetization (which is still along the easy *x*‐axis of the waveguide) and thus exert no torque. After the rotation of the static magnetization toward the *y* axis is completed at ≈20 mT (see Figure 3d), efficient spin‐wave excitation takes place, as confirmed by further dynamic micromagnetic simulations.

The color plot of Figure 2a is essentially symmetric, apart from some small deviation at low fields which can be ascribed to small misalignments of the conduit with respect to the field and from a general higher intensity of the oscillations for positive applied fields due to the non‐reciprocal behavior of DE spin waves.^[^ ^34^ ^]^ Interestingly enough, however, there are two minima in the color maps of Figure 2a, at −12 and +18 mT, delimiting a region around Ha = 0 where the iso‐phase lines display a slope with different sign with respect to that seen at larger applied fields. This is again the typical hard‐axis behavior indicating the reorientation of the sample magnetization as discussed in Figure 3. Restricting our attention to positive fields, the region with positive slope at large fields (µ_0_Ha >20 mT) can be associated to well‐defined DE modes with positive group velocity, while that corresponding to 0 < µ_0_Ha < 20 mT reflects other BV‐like modes with negative group velocity.

To gain a deeper insight into SW propagation in the conduits, in Figure 2b we report the traces of Im(S_12_) for some selected applied fields (continuous lines), together with a fit (dashed lines) carried out with the following function:

(1) $$Im\left(S_{12}\left(\omega \right)\right)=S\cdot \sin\left(k\left(\omega \right)r+\phi \right)\cdot e^{-r/L_{att}}\cdot \eta \left(k\left(\omega \right)\right)$$

where *S* is the amplitude of the oscillating term sin(k(ω)r+φ) describing SW propagation, k(ω) is the inverse function of the band dispersion for DE modes in a metallic stripe,^[^ ^35^, ^36^, ^37^ ^]^ *r* is the distance between input and output antennas, φ is a phase constant. The latter includes phase shifts introduced by the RF probe positioning, which in turn determines some variations in the location of reference planes, and VNA signal processing adding multiples of 2*π*. The function $e^{-r/L_{att}}$ takes into account SW attenuation which is also frequency dependent as the attenuation length L_att_ is given by the product of the relaxation time τ(k) and the group velocity v_g_(k). η(k) represents the antenna excitation efficiency, proportional to the spatial Fourier transform of the magnetic field produced by the antenna (see Section S3, Supporting Information) and set equal to zero for frequencies below the bottom of the SW band. The fit function of Equation (1) has been convoluted with a gaussian function with FWHM (200 MHz) corresponding to the experimental linewidth of the FMR curve for our CoFeB films.

The fit parameters are listed in **Table** **1**, while in Figure 2b we report for each curve the experimental applied field Ha (first number outside the brackets, in mT) and the bias field H_0_ used to obtain the best fit (number within brackets). Notice that the effective field (He) used for the derivation of dispersion relations is the sum of H_0_ and of the demagnetizing field (µ_0_H_M_ = −6.3 mT) estimated by the micromagnetic simulation of the conduit under action of a transversely applied field which causes its saturation: He = H_0_ + H_M_. A nice fit is obtained above 20 mT, with H_0_ = Ha, in agreement with the fact that only above that field a clear DE configuration is set in the conduit, with M_0_ perpendicular to the direction of propagation. Below 20 mT, instead, we observe a different kind of oscillations that can be ascribed to hybrid modes (between well‐defined DE and backward volume (BV) modes) arising from a multidomain configuration with average M_0_ displaying a sizable longitudinal component (See Figure 3). This interpretation is fully consistent with the above discussion on the origin of the two minima observed in Figure 2a. The fit function of Equation (1) does not apply to this situation and, consequently, no value for the bias field H_0_ can be retrieved from the fit.

**Table 1:** Micromagnetic and geometry parameters used from the fit of Im(S_12_) signals measured on reference CoFeB conduits not surrounded by MFC and SmCo permanent magnets.

Ms [A m^−1^]	A [J m^−1^]	α	γ/2π [GHz/T]	w_CoFeB_ [µm]	t_CoFeB_ [nm]	w_antenna_ [µm]	t_antenna_ [nm]	t_spacer_ [um]
1.3·10^6^	18·10^−12^	0.0043	28	3.6	26.3	1	129	70

Figure 2c reports the impulse response s_12_(t) of reference conduits at the same fields considered in Figure 2b. Starting from S_12_(f) signals taken at constant input power (−10 dBm) using a harmonic frequency grid [δf, 2δf, 3δf, …, Nδf], with δf = 4 MHz and N = 10 000 frequency points in each sweep, s_12_(t) is obtained as the Fourier transformation of the Hermitian completed $\tilde{S}$_12_(f) function:

(2) $$s_{12}\;\left(t>0\right)=F\left({\tilde{S}}_{12}\left(f\right)\right)$$

Mathematically this represents the response to an impulse corresponding to a normalized *sinc* function with duration (δt between the first zeros) of 2/f_max_ = 2/(Nδf) = 50 ps. For Ha >20 mT a wavepacket is clearly distinguishable, whose arrival time at the output RF antenna increases from ≈0.4 to 0.6 ns with the applied field, as expected because the group velocity for DE SW decreases with the applied field. At 20 mT additional oscillations are seen at larger arrival time, after the main wave packet, thus signaling contributions from other modes than the pure DE one. Below 20 mT no clear response is seen, apart from a residual signal close to t = 0 s which can be ascribed to a non‐perfect cancellation of the direct electromagnetic coupling between antennas via time‐gating (see Section S3, Supporting Information).

Overall, the analysis of reference conduits provided a way for checking the consistency of our methodology and calibrate our fitting procedure to retrieve quantitative information from the fit of data taken on devices including MFCs and permanent magnets.

VNA measurements performed on a fully integrated device, with a separation distance *D* = 12 µm between the SmCo magnet and the Py‐based MFC, are presented in Figure 2d–f. To ensure complete saturation of the SmCo hard phases and maximize the internal bias field, the samples corresponding to Figure 2d–i were first magnetized using a 5.4 T magnetic pulse, as described in the Methods section. Indeed, data acquired from samples magnetized at 2 T using a quasi‐static field (see Section S4, Supporting Information) indicate a reduction in the on‐chip bias field, particularly at larger *D* values. Conversely, for smaller D, the difference between samples magnetized at 2 and 5.4 T is less pronounced. This suggests that while an increase in remanent magnetization occurs, it is counterbalanced by a reduction in the gain of the MFCs, which become partially saturated due to their proximity to the SmCo magnets.

For devices with integrated magnets, the signal associated with SWs is obtained by subtracting a reference signal measured at an external field that compensates for the internal field generated by the magnets, ensuring that the actual bias field experienced by the magnonic conduit in that condition is effectively null. To identify this compensation point, a field sweep from +100 to −100 mT was first performed, and the S_12_ signal taken at the field where no oscillations due to SW propagation were detected was used as reference. In the case of *D* = 12 µm (panels d,e,f in Figure 2) this occurred at −5 mT, corresponding to the center of the excitation gap between the two branches seen in Figure 2d.

The Im(S_12_) color map upon background subtraction shown in Figure 2d, corresponding to *D* = 12 µm, clearly illustrates the influence of the bias field produced by the combination of permanent magnets and MFCs. The asymmetry of the map with respect to Ha = 0 mT confirms the presence of a positive bias field H₀ on the CoFeB waveguide, while the squeezing along the Ha axis (evident from the smaller separation between the two minima and the larger slope of the iso‐phase lines) clearly reflects the gain of the MFC. The bias field can thus be estimated by multiplying the observed shift of ≈5 mT by the MFC gain (*G* = 2.4), yielding H₀ ≈11 mT.

The oscillations of Im(S_12_) both in the frequency and time domain are shown in Figure 2e,g, respectively, for selected values of the applied field µ_0_Ha (in mT), indicated by numbers outside brackets. The corresponding bias field H_0_, extracted from fitting the imaginary part of S_12_(f), is reported in brackets. Notably, unlike the case of reference conduits in Figure 2b, oscillations persist even at µ_0_Ha = 0 mT, demonstrating that SW propagation is sustained without the need for an external electromagnet. However, at zero applied field, the oscillations have a drastic reduction in intensity, making a reliable fit challenging. This region corresponds to one of the minima in the color map (Figure 2d), where a pure Damon‐Eshbach (DE) SW model is not applicable. In the time domain (Figure 2e), DE wave packets propagate well down to 5 mT, but at µ_0_Ha = 0 mT, the time response broadens, suggesting an alternative band dispersion mechanism.

A nominal DE propagation in the CoFeB conduit is observed at zero applied external field in devices with *D* = 0 µm, where the SmCo magnets are in direct contact with the MFCs (Figure 2g–i). The Im(S_12_) color map in Figure 2g is shifted by ≈9 mT toward negative fields, while the spacing between the two minima remains comparable across devices, as the MFC gain is largely unaffected by the non‐uniform bias field from the SmCo magnets.

A key distinction in this configuration is that, starting from high positive fields, the iso‐phase lines in the color plot exhibit a monotonic decrease, crossing the zero‐field axis above 2 GHz. This indicates the establishment of a well‐defined DE configuration without the application of any external bias field. Fitting the Im(S_12_(f)) functions in Figure 2h, taken at the value of Ha (in mT) reported out of the bracket close to each curve, the bias field at the conduit region flanked by the antenna can be extracted (values in brackets, in mT). For µ₀Ha = 0 mT (bottom blue curve in Figure 2h), the estimated bias field is µ₀H₀ = 18 mT, in nice agreement with the ≈9 mT shift in the color map if one considers that the MFC gain G estimated form MOKE is 2.4 ± 0.3.

Interestingly, wave packets are also observed in the impulse response at µ_0_Ha = 0 mT, confirming that this device represents a first demonstration of a fully integrated stand‐alone magnonic device suitable for the processing of RF signals.

### 2.3 Micro‐Brillouin Light Scattering (BLS) Investigation

**Figure 4:**
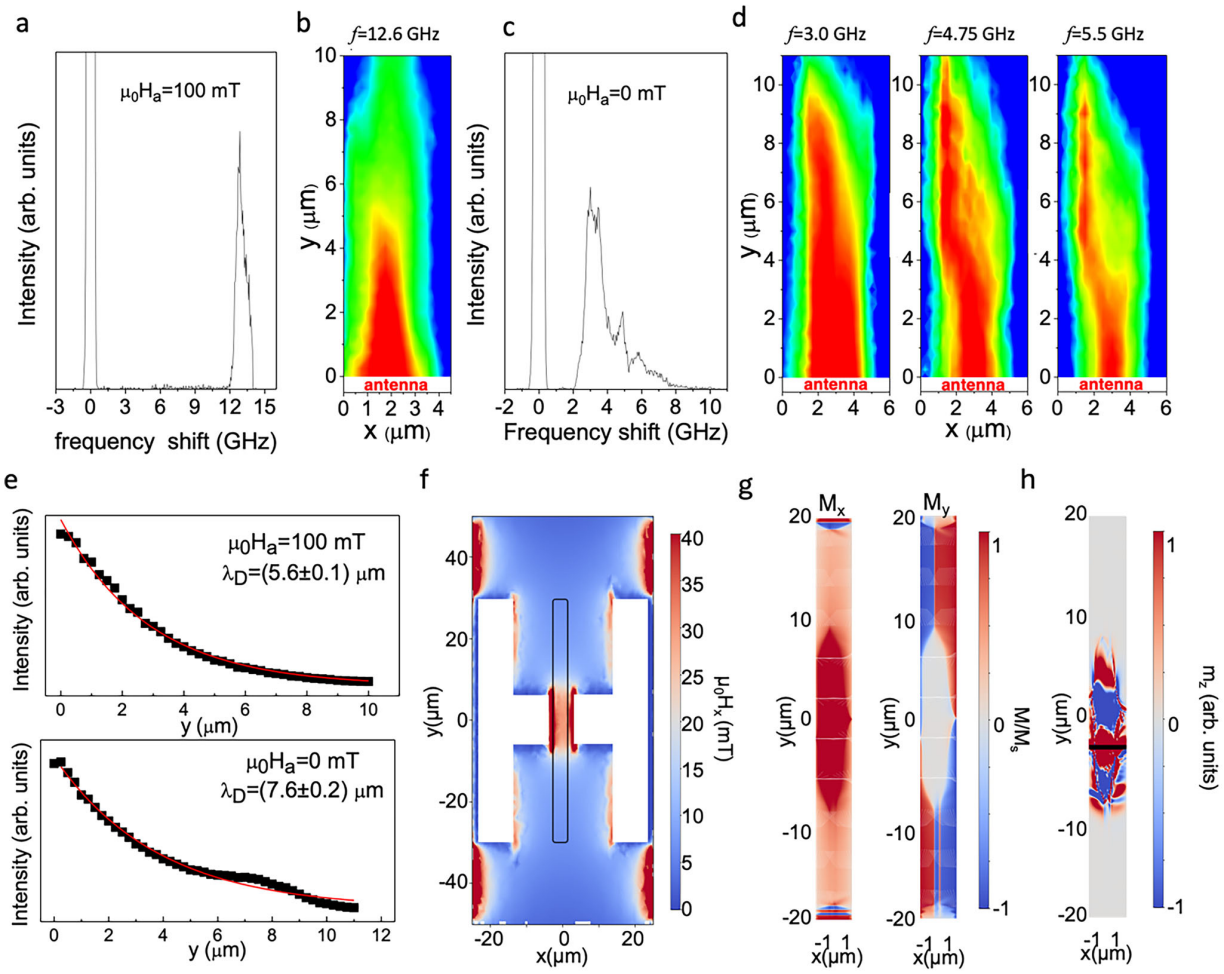
a,c) Spin wave spectra recorded by micro‐BLS at µ_0_H_a_ = 100 mT and µ_0_H_a_ = 0 mT, respectively. b) 2D map of the BLS intensity recorded at a frequency of 12.6 GHz excited by the inductive antenna. An external field µ_0_H_a_ = 100 mT was applied along the short axis of the conduit. d) 2D maps of the BLS intensity recorded for the modes at frequencies of 3.0, 4.75, and 5.5 GHz, respectively at µ_0_H_a_ = 0 mT. e) Spin‐wave intensity (linear scale) as a function of the propagation distance y from the antenna for µ_0_H_a_ = 100 mT (top panel) and µ_0_H_a_ = 0 mT (bottom panel). Black points correspond to experimental data, whereas red lines show the exponential fit. f) Spatial profile of the bias field H_0,_ generated by the micromagnets, simulated using COMSOL Multiphysics for an asymmetric position of the CoFeB conduit relative to the center of the MFCs. T‐shaped MFCs are shown in white. g) Micromagnetic simulations of the static magnetization of the CoFeB. h) Simulated intensity profiles of the SW mode propagating at 4.7 GHz. The horizontal black line indicates the excitation region.

Spin wave propagation has been investigated by BLS in a device with *D* = 0 µm, where the SmCo magnets are slightly superposed to the MFCs. The sample underwent a first magnetization up to only 2T in a VSM electromagnet, using a quasi‐static process. The sample's layout (**Figure** **4**a) is the same of the devices studied by broadband spectroscopy (reported in Figure 1a), apart from the positioning of the second antenna (not used in the BLS experiments) which is placed out of the region corresponding to the horizontal arm of the T‐shaped MFC. First, we analyzed the spin wave propagation in a well‐defined DE configuration, with M_0_ perpendicular to the spin wave wavevector, under an external magnetic field µ_0_Ha = 100 mT along the short axis of the waveguide.

To characterize the spin waves modes excited by the antenna in the CoFeB waveguide, the spin wave intensity was measured as a function of the excitation frequency in the range between 6 GHz and 16 GHz, at the center of the region flanked by the horizontal arm of the MFCs. As it can be seen in Figure 4b the BLS spectrum shows an intense peak at ≈12.6 GHz. Figure 4c shows a 2D map of the spin wave intensity, recorded at fixed frequency, over an area of ≈4.5 × 10 µm^2^ with a 250 nm step size. As it can be seen this mode is characterized by an almost uniform spatial profile across the width of the waveguide, as expected for the fundamental mode of a transversally magnetized conduit.

**Figure 5:**
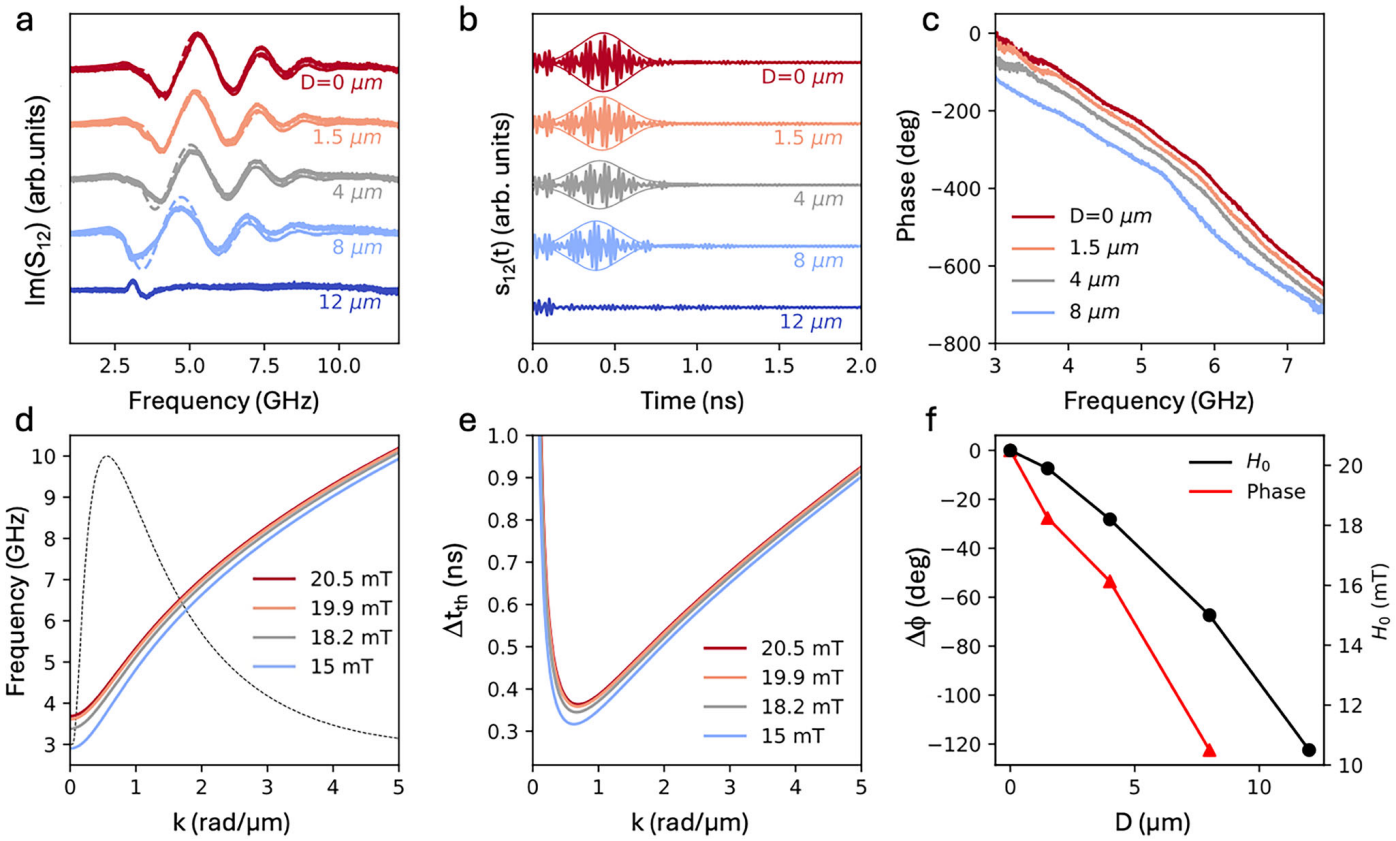
a). Im(S_12_) curves measured on devices with different values of *D* at Ha = 0 (when using them as standalone devices without any applied magnetic field). b) Impulse response of the same standalone devices from the time‐domain analysis of S_12_(f) scattering parameters. c) Relative phase of S_12_(f) for devices with various *D* values. d) Continuous lines: calculated band dispersions of DE spin waves for bias field values H_0_ reported in the legend and corresponding to *D* = 0, 1.5, 4, 8 µm. Dashed line: β(k) function defining the bandwidth of the magnonic device for the case of *D* = 0  microns. e) Estimated propagation time of wavepackets from the two antennas according to the formula Δt_th_ = r/v_g_, where v_g_ is the group velocity calculated from the band dispersions of panel 5d and r is the distance between the input and output antennas. f) Right scale: bias field µ_0_H0 estimated from the fit of curves in panel 5a. Left scale: phase shift introduced in magnonic devices with different *D* at 6 GHz.

Then we studied spin wave propagation at zero external applied field µ_0_Ha = 0 mT, following the same methodology. Figure 4e reports the BLS spectrum of spin waves excited by the antenna in the range between 2 and 8 GHz measured at the center of the region flanked by the horizontal arm of the MFCs. In addition to an intense peak at ≈3 GHz, one can observe two modes having a lower intensity at ≈4.75 and 5.5 GHz. Figure 4e shows 2D maps of the spin wave intensity acquired at fixed frequency for the three observed modes as a function of the distance from the antenna over an area of ≈6 × 11 µm^2^ with a 250 nm step size (Figure 4f). We found that the most intense mode is characterized by an almost uniform spatial profile across the width of the waveguide, while the other two modes exhibit a more complex spatial profile. One can also observe that all the modes tend to localize close to the left border of the waveguide, as the distance from the antenna increases. This behavior, which has not been observed in the measurements at µ_0_Ha = 100 mT, can be ascribed to a little misalignment of the waveguide with respect to the center of MFCs occurred during the device fabrication, causing a small inhomogeneity of the bias field H_0_ which becomes negligible when an intense field is applied. To corroborate this interpretation, we simulated the propagation of a SW mode excited at a frequency of 4.7 GHz, for a zero external applied field (µ_0_Ha = 0 mT), for a misalignment of ≈0.7 µm of the CoFeB conduit with respect to the center of the MFCs which corresponds to the real geometry of the device investigated by BLS. Micromagnetic simulations were performed using the profile of the bias field H_0_, calculated by COMSOL Multiphysics (Figure 4d). As it can be seen, the small inhomogeneity of the field along the x‐direction causes a distortion of the static magnetization (Figure 4e), which is better aligned along the x‐direction in the region where the bias field is more intense. Consequently, the SW mode tends to localize in this area of the conduit where the DE configuration is better defined, (Figure 4f) in agreement with the experimental results. As shown in Section S6 (Supporting Information) this effect disappears when the SW mode propagates in a CoFeB conduit is symmetrically placed between the MFCs. Comparing the frequency measured for the uniform mode with the dispersion relation of DE modes calculated the analytical model for SW propagation in metallic ferromagnetic stripes for a CoFeB conduit transversely magnetized (Figure 5f) we estimated a bias field produced by the magnet of about µ_0_H_0_ = 15 mT.^[^ ^37^ ^]^ This value is slightly smaller than the 18 mT estimated from VNA measurements in Figure 2g from the same device magnetized at 5.4T, but in good agreement with the value (16 mT) estimated by VNA upon magnetization up to just 2T (See Section S4, Supporting Information), as in case of BLS experiments. This estimation is also consistent with the appearing of the two additional slightly intense modes reflecting some inhomogeneities of the static magnetic configuration. Micromagnetic simulations, indeed, indicate that a single domain configuration is attained only for an applied transverse field larger than 20 mT (Figure 3).

Finally, it is interesting to note that at zero applied field the fundamental mode exhibits a propagation distance like that observed for a well‐defined DE geometry stabilized by the application of an intense field. In particular, the spin wave decay length has been estimated from the fit of the micro‐BLS intensity profile taken in the whole conduit, by using the equation

(3) $$I\;\left(y\right)=I_{1}\;\exp\left(\frac{-2y}{\lambda_{D}}\right)+I_{0}$$

where 𝑦 is the position along the waveguide, 𝐼_1_ the SW intensity at the antenna position, and I_0_ the offset baseline due to noise and detector dark count effects. As shown in Figure 4d,g, we found that the decay lengths are comparable, assuming the values λ_*D*_ = (7.6 ± 0.2)  µm and λ_*D*_ = (5.6 ± 0.1)  µm for µ_0_Ha = 0 mT and µ_0_Ha = 100 mT, respectively. This finding confirms that the bias field H_0_ provided by the micromagnets is strong enough to allow a good spin wave propagation in the DE configuration even at zero applied field, in agreement with the VNA spectroscopy results.

### 2.4 Functional Properties of Standalone Devices

To assess the potential of our devices we investigated in more detail the transmission of SW between the input and output RF antennas in standalone devices (i.e., without any applied external magnetic field), as a function of the distance *D* between the MFC and the permanent micromagnets. **Figure** **5**a shows Im(S_12_) curves versus frequency taken at zero applied field for *D* = 0, 1.5, 4, 8, 12 µm. For *D* from 0 to 8 µm, we observe well‐defined oscillations with a low‐frequency edge (corresponding to the FMR limit) which shifts toward lower frequency when increasing D, as expected due to the decrease of the bias field H_0_. In this range of distances, the experimental curves can be nicely fitted using the model described above for DE spin waves. This is no more possible at *D* = 12 µm, where the bias field is not high enough to overcome the anisotropy field arising from shape anisotropy of the CoFeB conduit, and a pure DE configuration cannot be achieved. The bias field H_0_ in the central region of the conduit for the various values of D, as derived from the fit of S_12_(f) signals, is shown in Figure 5f. Notice that these bias field values are slightly higher than those of Figure 2. This is because the detailed 2D maps of Figure 2 were measured one month after curves of Figure 5 and we observed in the meantime a degradation of the SmCo micromagnets which is due to a non‐optimized fabrication process used in this proof‐of‐concept device (See Section S5, Supporting Information for details and strategies to minimize it). Coming to the internally generated bias fields observed in this set of measurements on “fresh samples” (Figure 5f), a sizable variation from 20.5 to 11 mT is observed. This provides an easy way to tune the basic functional properties of this proof‐of‐concept device, namely the time delay and the phase shift of RF signals between the input and output antennas. Figure 5b shows the impulse‐response s_12_(t) of each device obtained from the analysis in the time‐domain of the S_12_(f) curves of Figure 5f. The experimental propagation time of the SW wave packet from the two antennas (∆t_exp_), which represents the key performance indicator for a device operated as a time‐delay line, varies between 370 and 450 ps when increasing *D* from 0 to 8 µm.

If we consider an applied signal at 6 GHz, i.e., in the middle of the whole frequency band of our prototypes, the measured 180 ps time delay range (from 370 to 450 ps) is more than one period of the signal. This is the minimum value to be used in delay lines and is placed in the lower range compared to examples reported in scientific publications or corresponding to commercial components (e.g., ADAR4002 from Analog Devices).^[^ ^38^, ^39^ ^]^

To assess the reliability of this analysis, we carried out a theoretical estimation of the propagation time in our conduits. In panel 5d we show the band dispersion of DE modes (continuous lines) according to the analytical model for SWs in ferromagnetic metallic stripes,^[^ ^36^ ^]^ for values of applied fields corresponding to the H_0_ from our analysis of S_12_(f) in standalone devices at various *D* (see panel 5f), together with the product of the antenna efficiency and the exponential attenuation (bounded by the FMR), expressed as β(k) = η(k)·exp(−r/L_att_(k)) for the case *D* = 0 (dashed line). It is quite evident that the frequency band in which we observe nice propagation of the RF signal is compatible with the low‐frequency bound imposed by the FMR and the high‐frequency one resulting from the loss of antenna efficiency at high wave vector. On the other hand, the downward shift of the bands at larger *D* (smaller bias field H_0_) is not rigid: close to the k values maximizing the antenna efficiency (≈0.6 rad µm^−1^) the slope increases at smaller fields. This is accompanied by an overall increase of the group velocity which translates into a decrease of the theoretical propagation time (Δt_th_ = r/v_g_) shown in panel 5e, in agreement with the trend of the experimental propagation time reported in Figure 5b.

On the other hand, comparing the absolute values of the experimental and theoretical propagation times in Figure 5b,e requires some preliminary considerations. According to the time‐domain analysis discussed above, the signals in Figure 5b correspond to the impulse response of an input pulse with a duration of 50 ps. This pulse represents an electromagnetic RF input signal composed of harmonic components with equal amplitude across the 0–40 GHz frequency range. However, this wide‐band signal is filtered by the transmission band of the magnonic conduit, which is limited between the ferromagnetic resonance (FMR) frequency and the highest frequency at which significant excitation efficiency of the antenna is observed. This filtering effect is evident in Figure 5d, where the black dashed line shows the function β(k) for a bias field µ_0_H₀ = 20.5 mT (D = 0 µm). This defines the effective bandwidth of the magnonic device. When extending this analysis across all values of D, we find that our devices have a spin wave transmission band ranging from ≈3 to 10 GHz, corresponding to an effective bandwidth Δf_e_ = 7 GHz for the input pulse traveling through the magnonic conduit. This translates into a longer effective duration of the input pulse, estimated to be ≈2/Δf_e_ ≈ 280 ps, which aligns well with the duration of the main wave packets observed in Figure 5b, in agreement with the fact that the pulse duration is also influenced by band dispersion. In contrast, when we estimate the theoretical propagation time Δt_th_ as the ratio between the distance between the two antennas (r) and the group velocity (v_g_), we are considering a much narrower (broader) wave packet in the frequency (time) domain with bandwidth Δf_k_ <<Δf_e_, centred at the frequency where v_g_ is calculated.

Despite the shape and width of the corresponding pulses is different, the propagation times observed in Figure 5b are consistent with the theoretical ones shown in Figure 5e if we restrict our attention to the region around the peak (≈0.6 rad µm^−1^) of the function β(k), which is the product of antenna efficiency and exponential attenuation of SWs (see Equation 1). In fact, we expect that the most significant contribution to the wave packets in Figure 5b arises from SW components around this value, where the calculated propagation times are on the order of 0.4 ns (Figure 5e), in good agreement with the position of the maximum of the envelope of waveforms of Figure 5b.

Considering the device as a “phase shifter,” we focus on evaluating the phase change induced by spin wave (SW) propagation between the antennas. This phase change can be estimated from the phase of the scattering parameter S_12_(f), as shown in Figure 5c within the 3–7.5 GHz range for values of *D* = 0, 1.5, 4, 8 µm. The phase of S_12_ at 3 GHz for *D* = 0 µm is used as a reference (the procedure for extracting relative phases from S_12_(f) measured on different devices is detailed in the methods section). First, we observe a monotonic decrease of the phase as a function of frequency, which is expected for the propagation of Damon‐Eshbach (DE) modes. More intriguingly, the phase shift at a fixed frequency can be tuned by ≈120 degrees by varying D, which corresponds to a significant change in the bias field H_0_, as previously demonstrated.

We would like to stress here that our work aims at disclosing a new technology platform based on the integration of multiple functional materials for the implementation of standalone magnonic devices. No optimization of the device functionality has been carried out for making them real RF components with well‐defined functionality. In particular, they have not been specifically designed to behave like True Time Delay components (with adjustable time delay which is constant within the frequency band of interest) or phase shifters (with constant phase shift within the frequency band). However, the order of magnitude of the tunable time delay (about one period of the RF signal) and of the phase shift (up to 120 degrees) indicates the potential of our technology platform to be used for the design and fabrication of realistic RF components.

Finally, let us discuss the temperature stability of our devices. According to the literature SmCo films have a Curie temperature (Tc) on the order of 700–800 °C, so that devices exploiting this material should not suffer from a sizable decrease of the stray field in a temperature range well below Tc.^[^ ^40^ ^]^ We tested the stability of our devices upon annealing at increasing temperatures, from 50 to 200 °C in steps of 50 °C. As reported in Section S8 (Supporting Information), we did not find any detectable variation of the scattering parameter S_12_(f) in zero‐applied field, thus indicating no detectable variation of the bias field produced by SmCo micromagnets. This analysis points out that our technology platform is suitable not only for consumer electronics but also for automotive and industrial applications which require stability up to 150 °C.

## 3 Discussion

To the best of our knowledge, the devices presented in this paper represent the first example of standalone magnonic devices with electric input/output fully integrated on silicon, capable of RF signal processing up to 8 GHz, and operating without external magnetic bias fields.

Each of them has a footprint of just 100 × 150 µm^2^ which is a record for a magnonic device and also for an integrated RF device, considering that with the current technology based on SAW the typical footprint of a filter is on the order of 1 mm^2^. This points to the huge potential of magnonics for the integration of RF components “beyond 6G.” Noteworthy, our devices are fabricated on‐silicon using a planar process suitable for low‐cost wafer‐scale production, as required for applications in consumer electronics.

Additionally, this paper demonstrates that our integrated magnonic devices are easily tunable, as the bias field H_0_ produced by the combination of permanent micromagnets and magnetic flux concentrators can be adjusted by varying the distance *D* between them. While the devices are not reconfigurable in operation (since the distance *D* is fixed during fabrication), the design is compatible with real‐time reconfigurability, aligning with the goals of the EU project MandMEMS which aims at combining MEMS and magnonics. In fact, the permanent magnets could be mounted on the movable parts of MEMS devices, allowing for continuous tuning of the distance D. Notably, displacements on the order of 10 µm, corresponding to the *D* values explored in this paper, are easily achievable with state‐of‐the‐art silicon‐based MEMS technology.

In this context, it is useful to summarize the performance of this first batch of demonstrators to assess the tunability range. From the data presented in Figure 5b, our devices could be used as time‐delay lines with tunable delays of up to 150 ps by varying *D* within the 0–8 µm range. Alternatively, they can be viewed as phase shifters with a tunable phase shift (Δϕ), as shown in Figure 5f, which illustrates the variation of Δϕ at 6 GHz as a function of the distance D. A tunable phase shift up to 120 degrees range was measured in this first batch of devices, while larger modulations can be easily envisioned.

To amplify the effect of the distance D, one can increase the strength of the stray magnetic field produced by the permanent magnets. This can be accomplished by either increasing the remanence magnetization of the SmCo pads (by playing with the phase composition and crystallization processes) or increasing the thickness of the SmCo layer. The stray field increases linearly with SmCo thickness for a fixed geometry, so doubling the thickness up to 2 µm could allow to achieve a bias field of 40 mT for small *D* and a phase shift of 2*π* when increasing the distance D, according to data obtained from reference conduits (see Figure 2b).

Other methods for biasing magnonic conduits and tuning SW transmission have been proposed, such as using current lines to generate variable magnetic bias fields or implementing magnetoelectric coupling. However, it is important to note that our approach, which relies on permanent magnets, is significantly more energy‐efficient. In fact, there is no energy consumption associated with generating the bias field, and modulation could be achieved using piezoelectric or capacitive actuations, which would require low power consumption. For comparison, generating a 20 mT bias field with a current line would require a current density of ≈4 × 10⁻⁶ A cm^−^^2^ in a wire with the same width as the magnonic conduit and 1 µm thickness, located 1 µm away from the conduit. This could result in substantial overheating of the device.

Finally, it should be noted that the signals presented in this paper were obtained by subtracting the reference signal, which comes from the direct electromagnetic coupling between the antennas. Due to the relatively high damping of CoFeB (5 × 10⁻^3^ in our films), the distance r between antennas ensuring a sizable magnitude of the SW signal at the receiver cannot exceed 10 µm. In our design, *r* = 5 µm, and the RF signal at the output antenna due to SWs is only 1% of the total signal. This makes direct application of these devices in real‐world scenarios impractical, although they are fully compatible with integration into consumer electronics products. A careful engineering and optimization process will be necessary to improve the technology readiness level and qualify these devices for RF signal processing applications, but this is beyond the scope of this work.

## 4 Conclusion

In this paper, we describe the realization of fully integrated and standalone magnonic devices on a silicon substrate. They are based on the combination of CoFeB conduits and an integrated magnetic assembly (magnetic flux concentrators plus permanent magnets) which internally generates the bias field needed to set a DE configuration for SW. The devices have a total footprint of 100 × 150 µm^2^ and feature an input and output antenna for RF signals processing in the 3–8 GHz range. We demonstrated that our proof‐of‐concept devices can operate without any external source of magnetic field, implementing some basic functionalities like that of a tunable time‐delay line or phase shifter. The tuning of the time‐delay (up to 150 ps) or phase shift (up to 120 deg at 6 GHz) is achieved by setting the distance *D* between the flux concentrators and the permanent magnets in the 0–8 µm range during fabrication. Future developments of this technology involve the realization of reconfigurable devices, by mounting the permanent magnets on the movable part of a MEMS to vary the distance *D* and thus the bias field.

## 5 Experimental Section

### Device Fabrication

The whole fabrication process is made of four steps. i) 1 µm thick SmCo permanent magnets (100 × 40 µm footprint) embedded in a Si(001) coupon (20 × 20 mm) are realized by sputtering deposition in a trench previously defined by reactive ion etching, with a mesoporous silica layer used for subsequent lift off. Details on the sputtering processes can be found in previous papers.^[^ ^27^, ^28^, ^30^ ^]^ Upon lift‐off an annealing at 650 °C for 30 min has been performed to promote the formation of the magnetic hard phase. (ii) 1 µm thick MFC made of a (Py(80)/Cr(5))_12_ multilayer (thickness in nm) are defined by sputtering and lift‐off. (iii) CoFeB waveguides are fabricated on the silicon substrate flanked by the assembly of MFC and permanent magnets by optical lithography, magnetron sputtering, and lift‐off. (iv) Finally, upon deposition of 70 nm of silica, RF antennas are realized by thermal evaporation and lift off of 100 nm of gold with 10 nm of Ti as an adhesion layer. Coupons are then cut into 10×10 mm samples containing arrays of devices with different layouts, and subsequently magnetized in a uniform field of 2 T provided by a conventional electromagnet available in the vibrating sample magnetometer setup. The samples used for the electrical characterization by VNA and reported in the main text have been magnetized with a pulse of 5.4 T provided by a magnetizer (Model *i Mag MicroMag* from Laboratorio Elettrofisico Engineering S.r.l.), ensuring a full saturation of the hardest SmCo_5_ phases typically found in the SmCo films.

### Micro‐MOKE

A home‐made microMOKE apparatus was employed in the longitudinal configuration, which involves applying a field parallel to the sample surface while simultaneously recording the reflected signal at a non‐normal angle to the surface to maximize the response associated with the in‐plane magnetization component. White p‐polarized light was used for illumination, while reflected light was analyzed via a polarizer in an s‐configuration to obtain the signal related to Kerr rotation, prior to image acquisition using a high‐sensitivity and high‐resolution charge‐coupled device (CCD) camera.

By analyzing the variation in magnetic contrast of a selected area of interest, local magnetization curves with micrometric resolution can be extracted (Figure 1c,d).

The images in Figure 1f–i were acquired using a 50x objective, enabling the capture of regions of ≈150 × 400 µm with a spatial resolution of ≈500 nm.

### VNA Spectroscopy

Broadband spectroscopy experiments were carried out using a home‐made RF probe station featuring a quadrupolar vectorial electromagnet suitable to produce in‐plane fields as high as 200 mT and a 43 GHz, 4 ports Vector Network Analyzer (R&S ZNA43).

### Brillouin Light Scattering

Micro‐BLS measurements were performed by focusing a single‐mode solid‐state laser (operating at a spectral line of 532 nm) at normal incidence onto the sample using an objective with a numerical aperture of 0.75, giving a spatial resolution of ≈250 nm. The inelastically scattered light was analyzed by means of a (3+3)‐pass tandem Fabry‐Perot interferometer. A nanopositioning stage allowed us to position the sample with a precision down to 10 nm on all three axes, and to perform spatial resolved scans moving the sample with respect to the objective. A spatially uniform magnetic field µ_0_H_A_ = 100 mT, provided by an electromagnet, was applied in the sample plane along the short axis of the waveguide. A DC/AC electrical probe station ranging from DC up to 20 GHz was used for spin‐wave excitation. The microwave power was set +0 dBm on the RF generator output.

### Simulations

Magnetic field simulations were performed using COMSOL Multiphysics 6.1 to optimize the system geometry. The material properties were incorporated using the Jiles‐Atherton model to reproduce the experimentally measured M(H) hysteresis loops of the (NiFe(80)/Cr(5))_12_ multistack and the CoFeB waveguide. The SmCo micromagnets were assumed to be fully saturated along the desired direction, with a magnetization value corresponding to the remanent magnetization measured via VSM. This assumption was justified by the fact that both the stray field and the applied field remain significantly lower than the coercive field required to alter the magnetization. The shape and dimensions of the field concentrators were iteratively modified and optimized to simultaneously enhance the magnetic field concentrated at the waveguide positions and improve sensitivity to variations in the micromagnets' positions, thereby increasing tunability. More details on the simulations can be found in the Supporting Information. The simulations of the equilibrium micromagnetic configuration and SW dispersion for different applied transverse magnetic fields were carried out using the mumax3 software.

## Conflict of Interest

The authors declare no conflict of interest.

## Supporting information



Supporting Information

Supplementary Video1

## Data Availability

Data available on Zenodo (https://zenodo.org/records/15827077) with DOI: 10.528/zenodo.15827077.
