# Supplementary material for: Standalone Integrated Magnonic Devices
Source: Adv Mater. 2025 Jul 23;37(40):2503493. doi: 10.1002/adma.202503493 (PMC12510288; doi:10.1002/adma.202503493)
Supplement: Supplementary file 1 — Supporting Information [file ADMA-37-2503493-s002.pdf]

ADVANCED MATERIALS

Supporting Information

for *Adv. Mater.*, DOI 10.1002/adma.202503493

Standalone Integrated Magnonic Devices

M. Cocconcelli, F. Maspero, A. Micelli, A. Toniato, A. Del Giacco, N. Pellizzi, A. E. Plaza, A. Cattoni, M. Madami, R. Silvani, C. Adelmann, A. A. Hamadeh, P. Pirro, S. Tacchi, F. Ciubotaru and R. Bertacco**

Supporting Information

Standalone integrated magnonic devices

M. Cocconcelli,* F. Maspero, A. Micelli, A. Toniato, A. Del Giacco, N. Pellizzi, A. E. Plaza, A. Cattoni, M. Madami, R. Silvani, A. A. Hamadeh, C. Adelmann, P. Pirro, S. Tacchi, F. Ciubotaru, R. Bertacco*

Section 1: Static simulations of the bias field from the micromagnets

Simulations were performed using COMSOL Multiphysics 6.1 to design and optimize the magnetic field configuration for the system under study.

The SmCo micromagnets are modeled as uniformly magnetized along the x-axis, with a magnetization value corresponding to their remanent magnetization, which was estimated experimentally via vibrating sample magnetometry. The magnets are designed to have an elongation along the direction orthogonal to the magnetization direction, to have an aspect ratio that maximizes the intensity of the x-component of the field and to ensure uniformity in the y-direction. The magnets are also designed to be as thick as possible within fabrication limits, which constrain the thickness to 1 micrometer.

To enhance field uniformity, two symmetrically placed magnets magnetized along the x-axis were employed. The intensity and uniformity of the magnetic field in the region between the magnets were found to strongly depend on their spacing, d. While reducing d increases the field intensity, it also compromises uniformity.

Magnetic flux concentrators (MFCs) were introduced to enhance both the field intensity and uniformity in the region of interest, where the magnonic conduit is located. Unlike configurations with single or paired magnets, the introduction of MFCs ensures that varying the spacing between the permanent magnet (PM) and the MFC alters only the field intensity without significantly affecting the field-line distribution.

Existing literature extensively examines the effects of MFC geometry on field amplification [Xiaoming Zhang et al, AIP Advances 1 December 2018; 8 (12): 125222]. However, previous studies primarily assume uniform external fields, whereas our work focuses on fields generated by permanent micromagnets operating at remanence. Therefore, the MFC geometry

was systematically optimized via simulations to maximize the amplification of the micromagnets' fields. Several shapes, including T-shaped, triangular, half-circle, wide-bar, and bar configurations, were tested. Among these, T-shaped concentrators showed superior performance for concentrating the non-uniform field produced by rectangular SmCo micromagnets and were further refined to maximize field concentration.

The objectives of this study are twofold: first, to optimize the magnets for maximizing field strength, and second, to optimize the MFC geometry for enhancing field amplification. Due to the interdependence of these components, a recursive design approach was employed. For instance, the MFC length dictated the minimum allowable spacing between the magnets, directly influencing the resulting field.

All relevant design parameters were systematically varied, starting from an initial configuration dictated by fabrication limitations, leading to the optimized values reported in Table S1 and Figure S1 a. The MFCs were designed to be as thick as possible with the thickness of Cr/MoNiFe multilayer limited to 1 μm. The MFCs consisted of alternating layers of Cr (5 nm) and MoNiFe (80 nm), a design choice intended to enhance magnetic permeability compared to bulk MoNiFe. This approach maximizes the gain of the concentrators.

The MFCs were positioned to minimize the gap between them, thereby increasing the field intensity while ensuring that their poles extended beyond the magnonic conduit width by a sufficient margin to accommodate lithographic resolution constraints during fabrication. The relative positioning between the MFCs and permanent magnets was also analyzed, as shown in Figure S1b. Reducing the gap between the magnets and the MFCs significantly enhanced the concentrated field strength. The simulations revealed that adjusting this gap could modulate the magnetic field by approximately 30 %, suggesting the potential for dynamic field tuning using MEMS actuators.

The T-shaped concentrator geometry and dimensions were selected to maximize the magnetic field over a region large enough to facilitate spin-wave excitation, propagation, and detection. The design also accounted for the minimum field of 18 mT required to fully bias a 3-micrometer-wide magnonic conduit made of CoFeB and set a DE configuration. The final optimized geometry, shown in Figure S1a, is expected to produce a field of 24 mT at the waveguide location, as seen in the field profiles shown in Figure S1b. The optimized geometrical parameters are reported in Table S1.

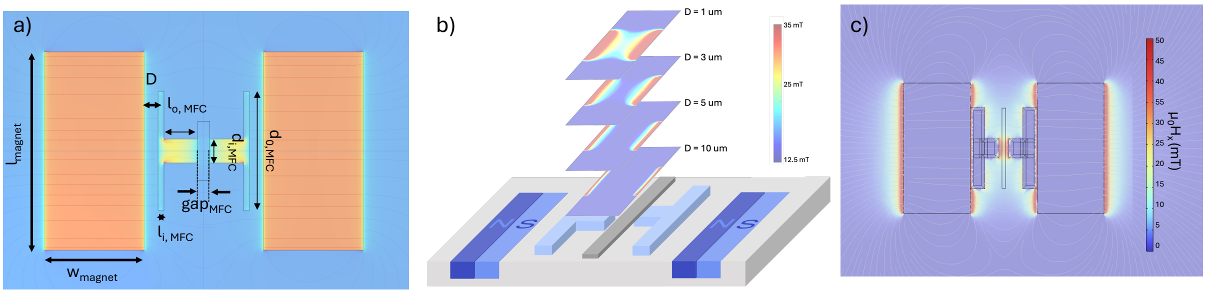

Figure S1: a) Layout (top view) of the system; b) Field concentrated by the MFCs in between their poles at varying distances (D) between the magnet and the MFC, c) field profile over a larger area for D = 1 µm.

Magnet length	Magnet width	$\mu_{r,py}$	M_r	Magnet thickness	$d_{i,MFC}$	$d_{o,MFC}$	$l_{tot,MFC}$	gap_{MFC}	MFC-magnet distance	Magnet z alignment with MFC
100 µm	50 µm	1000	1 T	1 µm	12 µm	60 µm	20 µm	6 µm	7 µm	Same plane

Table S1: Optimized parameters coming from COMSOL simulations and adopted for the design of devices investigated in the present paper.

Section 2: Comparison between MOKE loops taken at the center and edge of the conduits

Micro-Optical Kerr-Effect (MOKE) measurements were performed on the samples after the SmCo micromagnets were magnetized with a field of 2 T. Hysteresis loops of the CoFeB conduit were measured both at the center of the waveguide (between the poles of the magnetic flux concentrators (MFCs), where the field concentration is strongest), and at the edges of the conduit, where the concentration effect is weaker. The measurements were carried out for various distances between the magnets and the MFCs.

As shown in Figure S2, when the magnets are positioned close to the MFCs (D = 0), the hysteresis loop at the center of the conduit shows a significant shift, with a remanence value at zero external field comparable to the value at saturation. In contrast, the loop measured at the edge of the conduit displays a smaller overall shift and distinct jumps in the hysteresis curve. These jumps are attributed to Barkhausen noise, caused by the formation and movement of magnetic domains along the CoFeB conduit during magnetization reversal, as shown in Figure 1 in the main text. These domains propagate through the conduit and become pinned at regions where the field intensity varies, such as at the edges of the MFCs.

As the distance between the magnets and the MFCs increases, the ability of the MFCs to concentrate the magnetic field diminishes. Consequently, the loops show a smaller shift toward negative fields, and the hysteresis loops at the edge and center of the conduit become more similar. This is because the difference in the internal field between these two regions decreases as the distance increases.

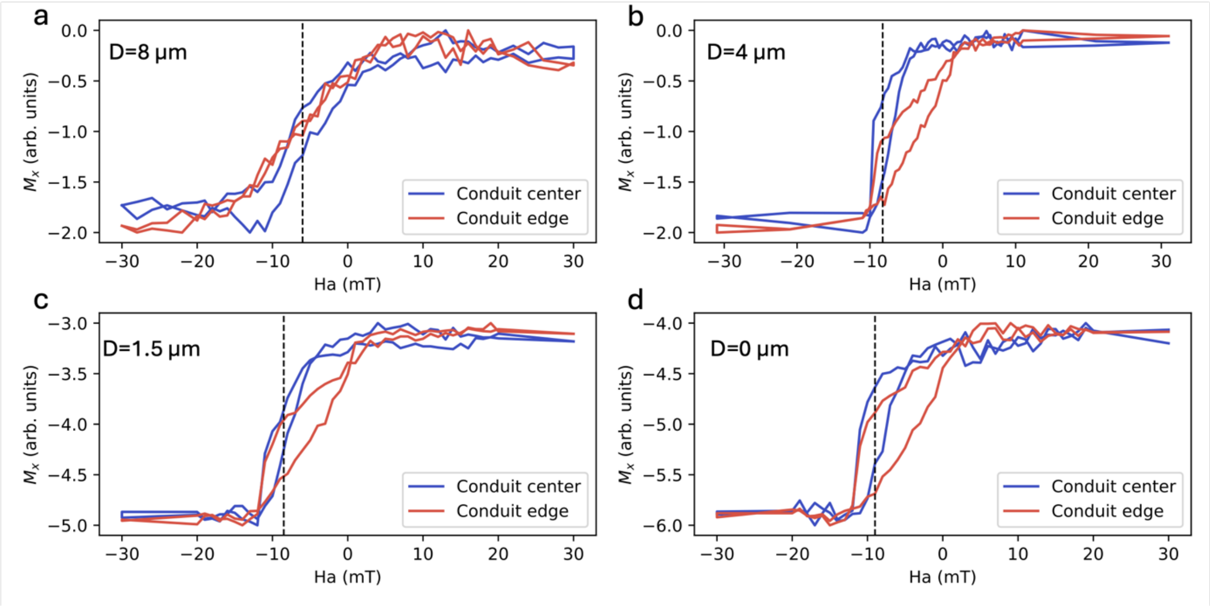

Figure S2: MOKE hysteresis loops measured at the center and at the edge of the conduit after magnetization of the SmCo micromagnets at 2T, in devices with different distance D between the MFC and the SmCo magnets.

Section 3: Data analysis of VNA spectra

3.1. VNA measurements and time gating

All-electrical measurements were performed on the samples using a Vector Network Analyzer (VNA), enabling the retrieval of the scattering matrix (S). We focused on the S_{12} parameter, which represents the complex transmission signal emitted from antenna RF_2 and received at antenna RF_1 (see Figure 1 in the main text).

The transmitted signal comprises both the spin-wave component and a background signal arising from electromagnetic coupling between the two antennas. Due to the small dimensions of the magnonic conduit and the intrinsic damping of CoFeB, the spin-wave contribution constitutes only a minor fraction of the total signal. To isolate the spin-wave signal, a reference subtraction method was employed to remove the electromagnetic noise. For the devices studied, the reference signal was obtained by applying an external magnetic field that

counteracted the on-chip field generated by the ensemble of magnetic materials. Under these conditions, the reference signal contained no spin-wave contribution.

However, even after reference subtraction, residual noise can persist due to temporal drift in the background or variations in the direct electromagnetic coupling as a function of the applied external field due to small displacements of the RF probes. Thus, to further reduce the noise, a time-gating procedure was applied. To enable time gating, measurements were conducted using a harmonic frequency comb (f, 2f, 3f, …), starting at 4 MHz and extending up to the maximum frequency achievable with the setup (40 GHz) for a total of 10000 points. The signal was then expanded to negative frequencies by filling in the complex conjugates of the positive frequency measurements and interpolating the value at 0 Hz from neighboring points. An inverse Fast Fourier Transform (FFT) was then performed to convert the frequency-domain signal into the time domain. The time-domain resolution was determined by the maximum frequency measured.

In the time domain, distinct signal packets corresponding to the spin-wave signal could be identified. A Hamming window function was applied to isolate the spin-wave signal, after which an FFT was performed to convert the signal back to the frequency domain. This procedure effectively filtered out noise, yielding a clean spin-wave signal, as shown in Figure S3.

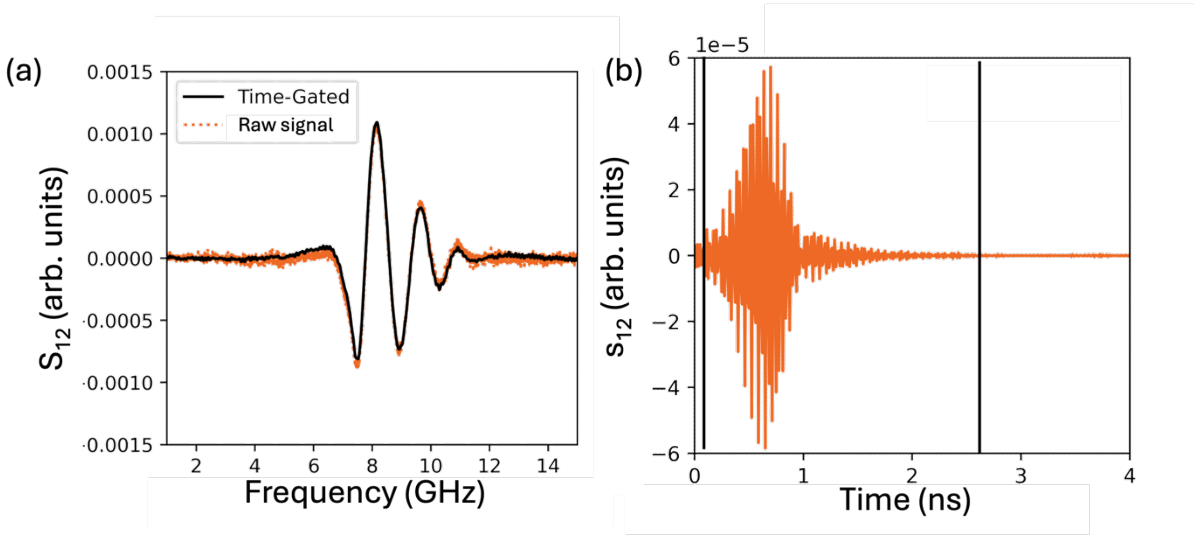

Figure S3: Signal from a reference device (CoFeB conduit without other on-chip magnetic elements) under an external magnetic field of 30 mT, shown in (a) the frequency domain and (b) the time domain. In panel (b), the black lines mark the boundaries of the window used for time gating. The gated signal corresponds to the black curve in panel (a).

This procedure is particularly effective when multiple modes are excited, as these modes may overlap within the same frequency band, making it challenging to distinguish between them. However, owing to their differing group velocities, the modes manifest as distinct packets in the time domain. By selectively applying time gating to these packets, it is possible to isolate the contributions of the individual modes in the frequency domain.

3.2. Model for fitting Im(S_{12})

To model the output of the Vector Network Analyzer (VNA), specifically the imaginary part of S_{12}, a one-dimensional model can be used, incorporating the dispersion relation and the antenna coupling efficiency [F. Ciubotaru et al, Appl. Phys. Lett. 109, 012403 (2016)]. The resulting expression is given by:

$$\mathrm{Im}(S_{12}(\omega)) = S \cdot \cos(k(\omega)r+\varphi) \cdot \exp(-r/L_{att}) \cdot \eta(\omega) \quad (1)$$

Where S is a coefficient, k(ω) represents the dispersion relation, r is the center-to-center distance between the antennas, φ is an additional phase introduced by the VNA, η(ω) is the antenna efficiency and L_{att} is the attenuation length of the spin wave.

The efficiency of the inductive antenna, η(k), is calculated by taking the Fourier transform of the magnetic field generated by the current flowing through the antenna. This field, evaluated at the position of the magnonic waveguide (separated from the antenna by a 70 nm SiO_2 interlayer), is computed considering the finite width and thickness of the antenna, which are 1 μm and 129 nm, respectively. The in-plane and out-of-plane components of the magnetic field are given by [R. Buschauer, Phys. Teach. 52, 413–414 (2014)]:

$$H_x(x,z) = -\frac{I}{8\pi ab}\left[(a-x)\left[\frac{1}{2}ln\left(\frac{(b-z)^2+(a-x)^2}{(a-x)^2+(-b-z)^2}\right)+\frac{b-z}{a-x}\cdot atan\left(\frac{a-x}{b-z}\right)-\frac{-b-z}{a-x}\cdot atan\left(\frac{a-x}{-b-z}\right)\right]-(-a-x)\left[\frac{1}{2}ln\left(\frac{(-a-x)^2+(b-z)^2}{(-a-x)^2+(-b-z)^2}\right)+\frac{b-z}{-a-x}\cdot atan\left(\frac{-a-x}{b-z}\right)-\frac{-b-z}{-a-x}\cdot atan\left(\frac{-a-x}{-b-z}\right)\right]\right]$$

$$H_z(x,z) = \frac{I}{8\pi ab}\left[(b-x)\left[\frac{1}{2}ln\left(\frac{(b-z)^2+(a-x)^2}{(-a-x)^2+(b-z)^2}\right)+\frac{a-x}{b-z}\cdot atan\left(\frac{b-z}{a-x}\right)-\frac{-a-x}{b-z}\cdot atan\left(\frac{b-z}{-a-x}\right)\right]-(-b-z)\left[\frac{1}{2}ln\left(\frac{(a-x)^2+(-b-z)^2}{(-a-x)^2+(-b-z)^2}\right)+\frac{a-x}{-b-z}\cdot atan\left(\frac{-b-z}{a-x}\right)-\frac{-a-x}{-b-z}\cdot atan\left(\frac{-b-z}{-a-x}\right)\right]\right]$$

Here, a and b represent the half-width and half-thickness of the antenna, respectively, while *x*, *z* are the transverse and out-of-plane coordinates of a reference system centered at the midpoint of the antenna. The Fourier transform of the field produced by the antenna provides η(k). This function, initially defined over the k-space determined by the spatial extent *x* used

in the calculation, is mapped through interpolation onto the k(ω) values corresponding to the dispersion relation. This mapping enables the evaluation of the antenna's excitation efficiency at each frequency of interest, η(ω).

The dispersion relation ω(k) for spin waves propagating in a transversally magnetized waveguide and its relaxation time, τ(k), are determined using a modified Kalinikos and Slavin model [M. P. Kostylev, G. Gubbiotti, J.-G. Hu, G. Carlotti, T. Ono, and R. L. Stamps, "Dipole-exchange propagating spin-wave modes in metallic ferromagnetic stripes", Phys. Rev. B 76, 054422 (2007)], using the material and geometrical parameters of the CoFeB waveguide reported in the main text and on the actual value of the external field.

The attenuation of spin waves is governed by the ratio between their attenuation length and the separation distance between the antennas. The attenuation length is calculated as the product of the relaxation time τ(k) and the group velocity $v_g(k)$. The group velocity is obtained from the derivative of the dispersion relation curve.

By combining these functions, the oscillatory behavior of the imaginary part of S_{12}, $Im(S_{12}(f))$, is reconstructed. To account for experimental broadening, the resulting signal is convoluted with a Gaussian function characterized by a full-width at half-maximum of 200 MHz, which corresponds to the experimental linewidth of the ferromagnetic resonance for the CoFeB films used in the study.

The model relies on several parameters, including the geometrical dimensions of the waveguide and antennas, the value of the applied external field, and the material properties of the CoFeB, as summarized in Table 1 of the main text.

To validate the accuracy of the model for our system, we applied it to reference samples. These consisted of CoFeB waveguides with known dimensions (without any additional magnetic materials nearby), placed in a uniform, externally applied field, and excited by stripline antennas identical to those used in the devices under study.

Fitting was performed using the model by fixing the external field and fine-tuning the magnetic and geometric parameters. Initial values for magnetic properties, such as saturation magnetization (Ms), damping constant (α), and exchange stiffness (A), were based on ferromagnetic resonance measurements conducted on continuous films of the same material. The initial geometric dimensions were obtained from scanning electron microcsopy. The best-fit parameters showed only minor deviations from the initial estimates, demonstrating both the accuracy of the model and its sensitivity to small variations in the magnetic and geometrical properties.

After confirming the reliability of the model, we applied it to fit the transmission signal recorded using the VNA for the devices under test. The goal was to estimate the internal magnetic field within the waveguide. For these fits, we kept the magnetic material parameters and all the other geometrical parameters fixed at the values emerging from the fit of the reference devices while considering the external field as free parameter for the fit. This approach produced accurate fits, as illustrated in Figure 2 of the main text, and provided the way to estimate the internal magnetic field considered in the modified Kalinikos and Slavin model, considering the finite width of conduit. From the internal bias field, the equivalent bias field produced by the micromagnets was determined by subtracting (adding in magnitude) the demagnetizing field, which was calculated using micromagnetic simulations and found to be 6 mT.

3.3. Phase signal from VNA measurements

The VNA allows for the extraction of the phase accumulated by spin waves as they propagate between two antennas. This is achieved by analyzing the phase component of the complex S_{12} matrix element. However, the phase signal may exhibit 360° discontinuities due to mathematical issues. Additionally, noise can introduce irregularities, resulting in spikes that complicate accurate signal interpretation.

In general, the phase of $S_{12}(f)$ corresponding to spin waves is expected to decrease monotonically when f increases, with a slope depending on the antenna separation and the dispersion relation, which allows for an easy differentiation from the background signal.

To accurately determine the accumulated phase for the various devices, the following procedure was implemented:

1. Time gating was applied to the S_{12} matrix element to reduce noise and isolate the spin-wave signal.
2. An offset correction was performed to eliminate 360 deg phase jumps, ensuring that the onset of the spin-wave signal started at 0 deg.

To further refine the estimation of phase variation and validate the accuracy of the procedure, the oscillatory behavior of the fitting function used to model the acquired signals was analyzed. The phase variation was then determined by calculating the phase differences between the curves corresponding to different devices, as shown in Figure S5. The phase difference among signals from devices with different D was then used to find the realative shift of the experimental curves $S_{12}(f)$ in order to compare them as in Figure 5c of the main text.

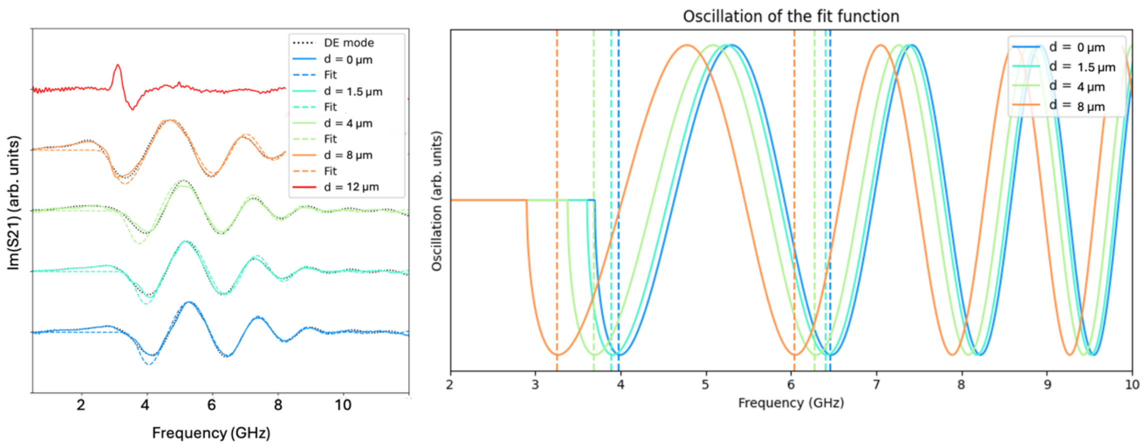

Figure S4: (a) Signal in the frequency domain (continuous line), gated signal corresponding to the main mode (black dotted lines) and fit function (colored dotted lines). (b) oscillating part of the fit signal, undamped, used for the estimation of the phase variation.

Section 4: VNA analysis on devices with SmCo magnets magnetized at 2T

VNA measurements were carried out upon magnetization of the SmCo micromagnets. Initially, the micromagnets were magnetized using a 2 T field (the maximum filed available in the laboratories of Politecnico di Milano) while only in a second step VNA measurements where refined upon magnetization at higher field (5.4 T) using a magnetizer kindly provided by Laboratorio Elettrofisico. While in Figure 2 and 5 of the main text we show VNA measurements after magnetization with 5T, here (Figure S4) we report data previously taken upon magnetization at 2 T, showing than in the latter case the bias field from the micromagnets was slightly lower. By applying the fitting procedure described earlier, also this case it was possible to estimate the field concentrated by the MFCs in the absence of an external field. For the case where the distance between the magnets and the MFCs was zero micrometers (D = 0 um), the bias field was found to be approximately 16 mT, in fair agreement with the shift of the symmetry axis of the 2D map of about 7.4 mT if one consider the gain of the MFC (G ~ 2.4, see main text). Notice the this bias field is slightly lower than the value of 18 mT of Figure 2 (1 month after magnetization at 5T, after some physical degradation of the SmCo magnets) and definitely lower than the 20.5 mT measured just a few days after magnetization at 5.4 T. Devices with greater distances between the magnets and the MFCs showed much weaker signals at 0 mT of applied field. The shapes of these signals suggest that the conduit configuration was not a pure DE one, with the magnetization vector displaying a longitudinal component which could account for the BV character of the $S_{12}(f)$

signals measured in this case. This is another indication that the magnetization at 5.4 T instead of 2T was effective in improving the bias field provided by the micromagnets.

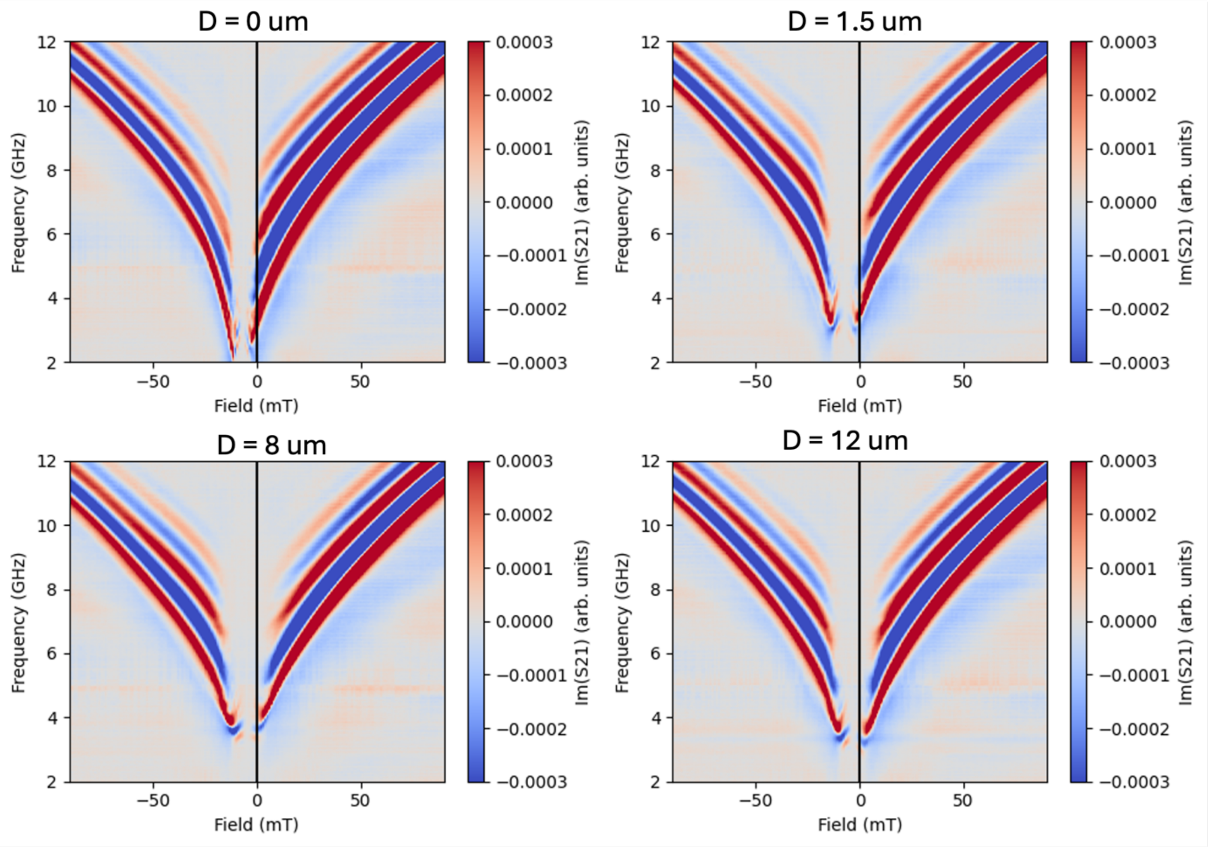

Figure S5: 2D color map of the oscillations related to the imaginary part of S_{12} vs frequency for various applied fields Ha, for devices with varying distance D between magnets and MFCs.

Section 5: Stability over time of SmCo micromagnets

The fabrication of SmCo micromagnets can, in principle, be achieved through either a lift-off process or ion beam etching of a pre-deposited film. However, when working with thick permanent magnet layers (e.g., > 500 nm), both approaches face significant challenges.

Lift-off is unfeasible due to the high temperatures required for SmCo deposition and post-deposition annealing, which are incompatible with conventional organic resists. On the other hand, ion beam etching of thick magnetic films is limited by redeposition effects, reducing pattern fidelity and etching efficiency [*Koplak, O. et al. (2024). Effect of patterning on SmCo micromagnets suitable for integration in microsystems. Journal of Magnetism and Magnetic Materials*].

To address these limitations, we developed a fabrication process based on an inorganic resist strategy using a mesoporous silica layer. The process flow is made of the following steps:

1. Spin-coating of a mesoporous silica layer onto a silicon substrate to act as a high-temperature-compatible resist,
2. Lithographic patterning of the micromagnet geometries and chemical etching of the silica to expose silicon in the desired regions,
3. Reactive ion etching (RIE) of the silicon to form pockets for SmCo deposition,
4. Deposition of a W/SmCo/W trilayer stack,
5. Lift-off via a 1% HF bath, leaving SmCo only in the predefined pockets,
6. Post-deposition annealing to crystallize SmCo and enhance magnetic properties.

As shown in Figure S6a, the undercut formed by the silica layer introduces a shadowing effect during deposition, resulting in a thinner W capping layer at the edges of the magnet. This effect is confirmed by EDX analysis (Figure S6g), which suggests a reduced W thickness at the borders.

Upon HF exposure during the lift-off, these edge regions become chemically vulnerable. EDX measurements (Figures S6e, S6f) show a well-defined edge for Sm, while Co and W maps display blurred edges along with the presence of fluorine. These observations suggest the formation of fluorine-based compounds and major chemical modifications, which compromise both the magnetic and mechanical integrity of the micromagnet at the edges. In some extreme cases - especially after prolonged HF exposure - visible cracking of the magnet edges can occur (see Figure S6b taken during the optimization of the process for the fabrication of SmCo micromagnets).

If the W capping layer is damaged not only at the edges but also within the interior of the micromagnet, similar degradation can occur throughout a larger portion of the structure. Therefore, damaged or incomplete W protection can allow progressive oxidation of the SmCo layer over time, as evidenced by the comparison between images taken immediately after annealing (Figure S6i) and five months later (Figure S6j). This degradation leads to a loss of magnetic field strength, directly impacting the on-chip bias field experienced by the active device layers.

However, when the W capping layer remains intact and uniform, as in the case of devices fabricated after a careful process optimization and discussed in the manuscript, degradation was limited to the edges. In these cases, a slight reduction in the stray magnetic field was observed during the first few weeks after fabrication and can be ascribed to the edge instability due to insufficient W capping. Although these affected regions are small, they are

located close to the flux concentrators and can produce a detectable variation in the local field. Importantly, this reduction stabilizes over time, and no further degradation was detected monitoring the bias field on the CoFeB waveguide, thus confirming the long-term stability of the magnet's functional properties.

To mitigate these issues in future fabrication runs, we plan to increase the thickness and improve the conformal deposition of the W capping layer, ensuring better protection at the magnet edges. Additionally, we aim to replace the chemical lift-off process with a dry, temperature-assisted lift-off, which should minimize edge damage and improve the overall structural and magnetic stability.

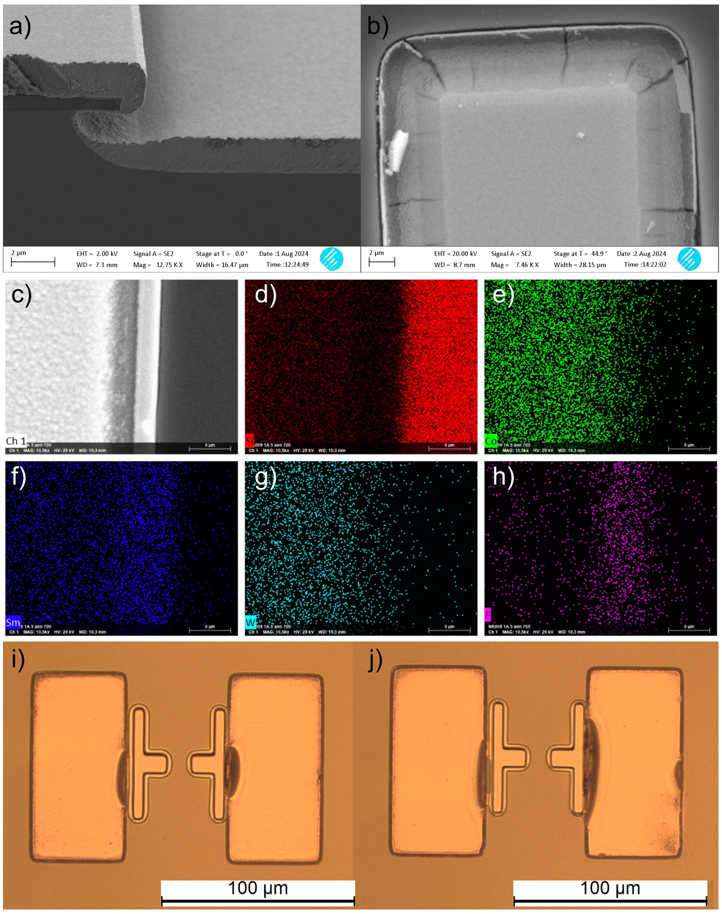

Figure S6: (a) SEM cross-section of a W/SmCo/W magnet deposited in Si trenches; (b) SEM image of a magnet showing edge degradation due to insufficient W capping; (c) SEM close-up of a magnet edge; (d–h) Energy Dispersive X-ray Spectroscopy (EDX) maps for (d) Si, (e) Co, (f) Sm, (g) W and (h) F, showing the same region as in (c); (i) optical image of a magnet

immediately after annealing; (j) optical image of the same magnet after five months, showing some degradation at the edges.

Section 6: SW propagation in a conduit symmetrically placed with respect to the magnetic flux concentrators

In this section we report the micromagnetic simulation of the SW propagation excited at a frequency of 4.7 GHz, for the CoFeB conduit symmetrically placed between the MFCs. Micromagnetic simulations were performed at a zero external applied field (μ_0Ha=0 mT), using the profile of the bias field H_0, calculated by COMSOL Multiphysics (Fig.S7a). Due to the quite uniform profile of the bias field along the x, the static magnetization is also characterized by a symmetric profile (Fig.S7b) and the SW mode propagates in the central region of the conduit. (Fig.S7c).

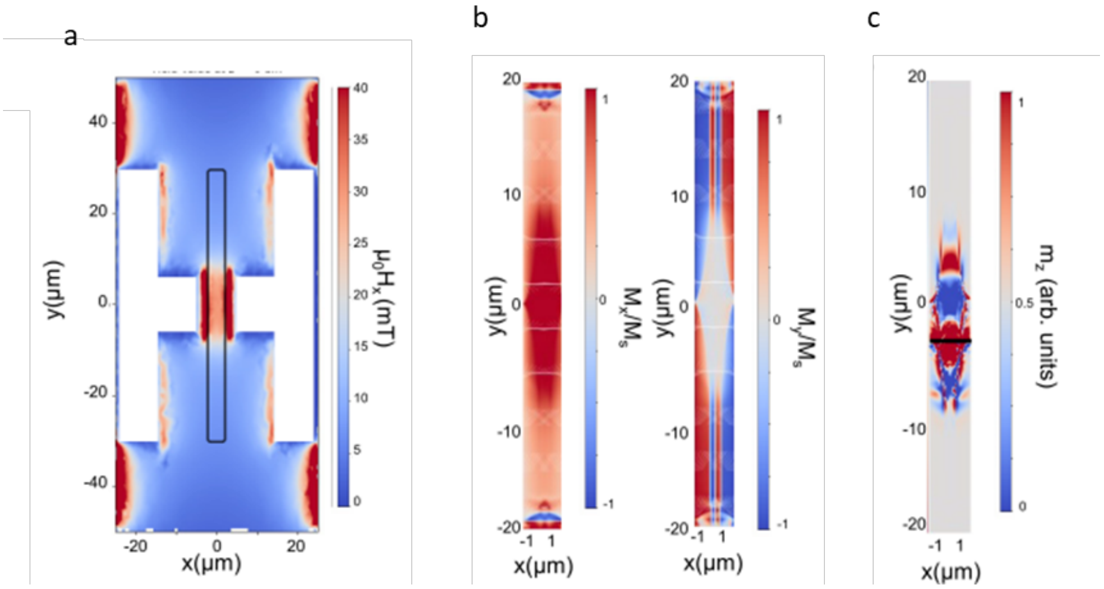

Figure S7 a) Spatial profile of the bias field H_0 generated by the micromagnets, simulated using COMSOL Multiphysics for a symmetric position of the CoFeB conduit relative to the center of the MFCs. T-shaped MFCs are shown in white. b) Micromagnetic simulations of the static magnetization of the CoFeB conduit. c) Simulated intensity profiles of the SW mode propagating at 8.0 GHz. Horizontal black line indicates the excitation region.

Section 8: Temperature stability

To assess the robustness of the device performance under thermal stress and to explore their potential for real-world applications, particularly in environments demanding high thermal stability, such as automotive electronics (typically requiring operation up to 150 °C), we conducted a temperature dependence study.

The thermal stability of the devices was tested through a controlled annealing procedure using a Rapid Thermal Annealer (UniTemp RTP-150-HV). The process followed these steps:

1. Chamber evacuated to a vacuum pressure of 10^{-3} hPa,
2. N_2 gas introduced at 5 ln/min until reaching 1 hPa pressure,
3. Rapid heating over 10 seconds to the target temperature,
4. Temperature held constant for 5 minutes.

This procedure was performed for annealing temperatures of 50, 100, 150, and 200 °C. Prior to and after each annealing step, optical microscopy images were captured to visually confirm that the permanent magnets remained structurally intact.

Subsequently, VNA measurements were carried out at each step, as shown in Figure S8, both at zero external field and under a reference magnetic field of 150 mT, according to the protocol detailed in the main text. Across the full temperature range, we observed no degradation in device performance. Zero-field propagation was consistently present, and the operational frequency band of the signal remained unchanged. This indicates that the magnetic properties, such as remanent magnetization and magnetization states of the SmCo magnets, were unaffected by thermal annealing up to 200 °C.

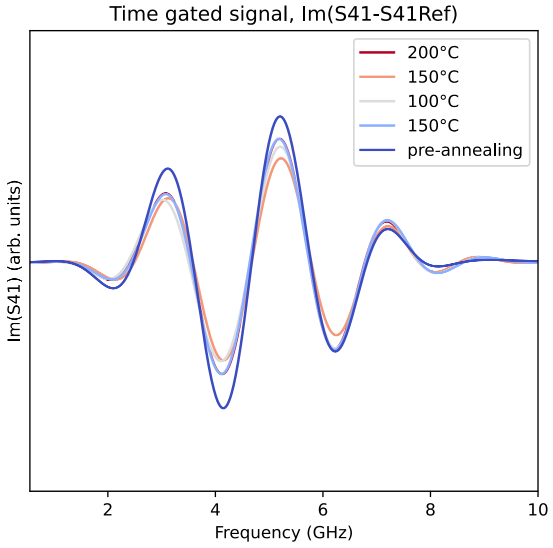

Figure S8: (a) Time gated signal in the frequency domain corresponding to the main propagation mode, measured before and after annealing at progressively higher temperatures.

A minor variation in the amplitude of the time-gated signal was noted, but this is not connected to the degradation of SmCo magnets, as the frequency behavior (related to the magnetic field) is unaffected. Most probably the upper limit for the temperature operational range is set not by SmCo but by the robustness of other elements like Au antennas and silica interlayers. These results suggest that the current magnet configuration provides sufficient thermal stability for many practical applications and also establish a reliable baseline for future efforts to optimize the magnetic material properties.
